# Differential expression of transcription factor- and further growth-related genes correlates with contrasting cluster architecture in *Vitis vinifera* ‘Pinot Noir’ and *Vitis* spp. genotypes

**DOI:** 10.1007/s00122-020-03667-0

**Published:** 2020-08-18

**Authors:** Robert Richter, Susanne Rossmann, Doreen Gabriel, Reinhard Töpfer, Klaus Theres, Eva Zyprian

**Affiliations:** 1 Federal Research Centre for Cultivated Plants, Institute for Grapevine Breeding Geilweilerhof, Julius Kühn Institute, 76833 Siebeldingen, Germany; 2 Department of Plant Breeding and Genetics, Max Planck Institute for Plant Breeding, Carl-von-Linné-Weg 10, 50829 Cologne, Germany; 3 Federal Research Centre for Cultivated Plants, Institute for Crop and Soil Science, Julius Kühn Institute, Bundesallee 58, 38116 Brunswick, Germany

## Abstract

Grapevine (*Vitis vinifera* L.) is an economically important crop that needs to comply with high quality standards for fruit, juice and wine production. Intense plant protection is required to avoid fungal damage. Grapevine cultivars with loose cluster architecture enable reducing protective treatments due to their enhanced resilience against fungal infections, such as *Botrytis cinerea*-induced gray mold. A recent study identified transcription factor gene *VvGRF4* as determinant of pedicel length, an important component of cluster architecture, in samples of two loose and two compact quasi-isogenic ‘Pinot Noir’ clones. Here, we extended the analysis to 12 differently clustered ‘Pinot Noir’ clones from five diverse clonal selection programs. Differential gene expression of these clones was studied in three different locations over three seasons. Two phenotypically opposite clones were grown at all three locations and served for standardization. Data were correlated with the phenotypic variation of cluster architecture sub-traits. A set of 14 genes with consistent expression differences between loosely and compactly clustered clones—independent from season and location—was newly identified. These genes have annotations related to cellular growth, cell division and auxin metabolism and include two more transcription factor genes, *PRE6* and *SEP1*-like. The differential expression of *VvGRF4* in relation to loose clusters was exclusively found in ‘Pinot Noir’ clones. Gene expression studies were further broadened to phenotypically contrasting F1 individuals of an interspecific cross and OIV reference varieties of loose cluster architecture. This investigation confirmed *PRE6* and six growth-related genes to show differential expression related to cluster architecture over genetically divergent backgrounds.

**Electronic supplementary material:**

The online version of this article (10.1007/s00122-020-03667-0) contains supplementary material, which is available to authorized users.

## Introduction

Grapevine (*Vitis vinifera* L.) is one of the most important fruit crops at global scale. The worldwide grape production reached 74 million tons in 2018 (OIV 2019). The world gross production value for grapes in 2016 was above 67.5 billion USD (FAOSTAT 2016). Regardless of the use as wine grapes, table grapes or dried fruits (raisins), only high-quality fruits are acceptable for marketing. Unfortunately, *V*. *vinifera* grapevine varieties are susceptible to several pathogens. Viticulture requires intense application of plant protection products (PPP) to meet the market’s demands. Fungicides are unavoidable to control the pathogens (Pertot et al. 2017) causing powdery mildew, *Erysiphe necator* (syn. *Uncinula necator,* (Schw.) Burr), downy mildew, *Plasmopara viticola* (Berk. & Curt) Berl. & de Toni) and *Botrytis cinerea* (teleomorph *Botryotinia fuckeliana* (de Bary) Whetzel), provoking gray mold. The use of PPP, irrespective of their inorganic (copper and sulfur) or synthetic origin, contributes to a decrease in biodiversity and raises consumers’ concerns (Keulemans et al. 2019). One strategy to reduce their use is the breeding of pathogen-resistant grapevine varieties, e.g., by introgression of genetically traceable resistance loci against *E*. *necator* and *P. viticola* from wild American or Asian *Vitis species* into *V*. *vinifera* quality cultivars. In the last years, several improved varieties with resistance traits against the mildews became available (Töpfer et al. 2011). However, for *B. cinerea*, there is only preliminary knowledge on a putative resistance locus (Sapkota et al. 2019). Current cultivar development focuses on the enforcement of physical barriers, e.g., a thick berry skin, a hydrophobic berry surface and loose cluster architecture, to increase resilience toward *B. cinerea* (Gabler et al. 2003; Herzog et al. 2015; Shavrukov et al. 2004). Within a loose grape cluster, improved ventilation accelerates the drying-off after rainfall or morning dew. Reduced humidity diminishes infections with fungal pathogens (Hed et al. 2009; Molitor et al. 2012). In addition, fungicide sprays can better spread into a loosely clustered bunch as compared to a compact one (Hed et al. 2010). The high physical stress arising in between the berries of compact clusters upon ripening provokes micro-cracks or even bursting of the berry skin (Becker and Knoche 2012; Smart and Robinson 1991). This problem is avoided in loosely clustered bunches. Moreover, there are less pronounced temperature gradients within loosely structured clusters as solar radiation can better reach the interior berries. This conveys more uniform fruit maturity (Pieri et al. 2016; Vail and Marois 1991). Overall, loose cluster architecture results in grapes with less *B. cinerea* infections and a better harmonized ripening process. It is a highly desired trait in grapevine breeding. Understanding its genetic basis would help to develop novel tools for efficient grapevine breeding and clonal selection.

Worldwide, several thousands of grapevine cultivars exist and are registered in data repositories, e.g., the ‘Vitis International Variety Catalogue’ (http://www.vivc.de; Maul 2019). A plethora of genetic diversity subsists and includes the gene pools of wine grapes and table grapes that show remarkable differences in berry and cluster architecture (Di Genova et al. 2014; Migicovsky et al. 2017). The variability of cluster density is characterized by OIV (Office International de la Vigne et du Vin, International Organisation of Vine and Wine, Paris, France) descriptors like OIV#204, and reference varieties for the scores of this descriptor are available (OIV 2015). However, despite the impressive genetic diversity, only 33 (*V. vinifera* L. subsp. *vinifera*) cultivars account for 50% of the totally used acreage for commercial production (OIV 2017). Promoted by the long cultivation time and large acreage covered with the predominant cultivars, somatic mutations causing intra-cultivar genetic variation are detectable and exploitable to select clonal variants (De Lorenzis et al. 2017). For example, about 500 different clones are available for ‘Pinot Noir’ (PN) (Forneck et al. 2009), a variety of high economic importance. Clonal selection programs in this cultivar identified phenotypic variants for relevant agronomic traits including cluster architecture. Apart from the mutation, these clones provide the opportunity to perform genomic diversity studies in a ‘pseudo’ near isogenic background (Blaich et al. 2007; Konradi et al. 2007). Phenotypic and genotypic diversity can further be uncovered in segregating cross populations intended for genetic mapping and development of trait-linked markers for breeding purposes. Several such populations for genetic tagging of cluster architecture traits were reported (Correa et al. 2014; Marguerit et al. 2009; Richter et al. 2019).

Bunch architecture is controlled by environmental and genetic factors (Döring et al. 2015; Tello and Ibáñez 2017). It is a complex trait composed of berry and stalk characteristics (Li et al. 2019; Richter et al. 2019; Rist et al. 2018). Some of these sub-traits are under genetic control as reported for berry size, berry volume and berry weight (Ban et al. 2016; Houel et al. 2015; Mejia et al. 2007; Tello et al. 2015), berry number (Dry et al. 2010; Fanizza et al. 2005) and other rachis sub-traits (Correa et al. 2014; Marguerit et al. 2009; Tello et al. 2016).

Intravarietal diversity in cluster architecture sub-traits of grapevine has been reported for only few cases, comprising clones of cultivars ‘Garnacha Tinta’, ‘Tempranillo’, ‘Aglianico’ and ‘Muscat of Alexandria’ (Grimplet et al. 2019, 2017; De Lorenzis et al. 2017). For ‘Albariño’ clones and for PN clones, the studies of Alonso-Villaverde et al. (2008) and Konrad et al. (2003) provided evidence that loosely clustered clones show reduced susceptibility to *B. cinerea.* PN is a member of the very old ‘Pinot’ family (Regner et al. 2000) and is used in viticulture for centuries. Presently, with an acreage of 115.000 ha, PN is among the top thirteen international varieties (OIV 2017). The ‘Pinot’ family accumulated a high number of somatic mutations and gave rise to a wide range of clones displaying divergent phenotypic features (different berry color, varying levels of acidity, different aroma compounds, different vigor and cluster architecture) (Forneck et al. 2009). Concerning cluster architecture (CA), the PN clones were classified into three categories, i.e., compactly clustered clones (CCC) with a dense arrangement of berries, loosely clustered clones (LCC) with berries not touching each other and loose clones with mixed berry size (MBC) producing bunches containing small and large berries at the same time (Bleyer 2001; Ruehl et al. 2004).

In PN, the gene *VvGRF4* was recently detected as a major component affecting inflorescence architecture (Rossmann et al. 2019). Two loosely clustered PN clones from the ‘Mariafeld’ selection line (M171) and the Geisenheim clonal selection program (Gm1-86) were compared to two compactly clustered clones (‘Frank Charisma’ and ‘Frank Classic’). This investigation included RNA-Seq analysis and revealed a mutation in the microRNA mi396 binding site of *VvGRF4*, a gene encoding a growth-promoting transcription factor. The mutation prevents down-regulation of the *VvGRF4* transcript, specifically in the LCC clones. Two mutated alleles were identified, one specific for M171 and the other one found in Gm1-86. Both operate in heterozygous state, lead to an enhancement of cell numbers in pedicels in the loose clusters and thus contribute to loose cluster architecture (Rossmann et al. 2019).

In this work, we explored the variation of cluster architecture in an extended set of twelve PN clones from five different selection lines and linked it to the differential transcriptional activity of genes selected from the previous RNA-Seq study. Two OIV reference varieties for loose cluster architecture and 16 selected F1 genotypes from a controlled cross (‘Calardis Musqué’ (formerly GF.GA-47-42) × ‘Villard Blanc’) segregating for cluster architecture traits (Richter et al. 2019) were included to broaden the analysis and validate the results. Besides *VvGRF4,* 14 more genes including two genes encoding additional transcription factors were found to be stably regulated in the quasi-isogenic ‘Pinot Noir’ plants, independent from their growth in different places and through several seasons. Out of these, a set of seven genes were found to be involved in the genetic regulation of cluster architecture sub-traits in different genetic backgrounds.

## Materials and methods

### Plant material

The *V. vinifera* variety ‘Pinot Noir’ (abbreviated PN, VIVC No. 9279) was investigated in 12 clones showing different cluster architecture. These comprised compactly clustered clones (CCCs), loosely clustered clones (LCCs) and clones bearing berries with mixed size (MBCs), the latter also resulting in loose clusters. The plants were distributed over three plantations in three German viticulture areas (Palatinate, Baden and Hesse) with partial overlap (Table 1). The vineyard in Palatinate is a trial field of Julius Kuehn Institute for Grapevine Breeding Geilweilerhof (JKI). The vineyards in Baden and Hesse originated from certified material and were managed by grapevine nurseries. All vineyards were submitted to regular visual monitoring for their phytosanitary state.

**Table 1:** Sampling schedules for 12 ‘Pinot Noir’ clones spread over three locations during two seasons for phenotyping

Cluster type	Sample	Abbreviation	Palatinate	Hesse	Baden
			BBCH 89	BBCH 89	BBCH 89
CCC	Frank Charisma	FkCH	10^a^	10^a^	10^a^
CCC	Frank Classic	FkCL	10^a^	10^a^	–
CCC	Entav 777	En777	–	10^a^	10^a^
Variable	Geisenheim 18	Gm18	–	10^b^	–
MBC	Geisenheim 20-13	Gm20-13	10^a^	10^a^	10^a^
MBC	Freiburg 1801	Fr1801	–	10^a^	10^b^
LCC	Geisenheim 1-86	Gm1-86	10^a^	10^a^	–
LCC	Freiburg 12-L	Fr12L	–	10^a^	10^a^
LCC	Freiburg 13-L	Fr13L	–	10^a^	10^a^
LCC	Weinsberg M1	WeM1	–	10^a^	–
LCC	Weinsberg M171	WeM171	10^a^	–	–
LCC	Weinsberg M242	WeM242	–	10^b^	–

For phenotyping of cluster traits, samples of ripe bunches at BBCH89 were taken with 10 replicates from randomly selected independent vines. The measurements of the PN clones ‘Frank Charisma’ (FkCH) and ‘Gm20-13,’ present at all three locations, enabled to model the environmental impact on cluster architecture sub-traits (Online resource 6 a, b and c)

– not available

^a^Biological samples taken in 2015 and 2016

^b^Biological samples taken in 2016

Trueness to type of the PN plants over all locations was confirmed with six SSR markers (VMC3a9, VMC5g7, VMC8g6, VrZAG79, VVMD32 and VVS2) described to monitor clonal variation in PN (Pelsy et al. 2010) in two snap samples per clone and location (44 samples in total, Online resource 1). SSR analysis was done as described (Zyprian et al. 2016).

The PN clones were well established (~ 20-year-old vines), and all grafted on the same rootstock (Kober 125AA, VIVC No. 12344). ‘Guyot pruning’ was applied throughout, and a vertical shoot position trellis system with 1.8–2.2 m^2^ space per vine was used. Vineyards in Baden and Hesse were maintained with integrated management. The PN field of JKI was managed according to organic farming rules (Online resource 2). All the plantations contained ample material of PN plants to permit random sampling from the individual clones. Samples were taken exclusively from plants without any symptom of infection or aberration from the typical clonal type of appearance. The OIV reference varieties for loose cluster architecture, ‘Uva Rara’ (VIVC No.12830) and ‘Prosecco’ (Prime name ‘Glera,’ VIVC No. 9741), were maintained in triplicates as part of the germplasm collection at JKI. The vines are grafted on rootstock ‘Selektion Oppenheim 4’ (SO4, VIVC 11473) and were planted in 2011. A set of 16 phenotypically extreme F1 genotypes (concerning the lengths of pedicels and rachises) from a controlled cross of ‘Calardis Musqué’ (synonym GF.GA-47-42, VIVC No. 4549) × ‘Villard Blanc’ (VIVC No. 13081) (Zyprian et al. 2016) used in this work (Table 2) were planted in eight replicates on rootstock SO4 at JKI in 2010. The OIV reference varieties and the F1 individuals underwent ‘Guyot pruning’ with approximately 10 buds remaining. They were grown in a vertical shoot position trellis system with 2 m (row) × 1 m (plant) spacing. An integrated pesticide spray program according to the best practice policies for viticulture (BMELV 2010) protects this plantation.

**Table 2:** Sampling schedules for phenotypically extreme F1 individuals of the cross ‘Calardis Musqué’ (formerly GF.GA-47-42) × ‘Villard Blanc’ grown in the Palatinate vineyard

Cluster type	Sample	Abbreviation	BBCH 89
Long pedicel	F1# 212, 294, 354, 380^a^	PEDmax	3–12^b^
Short pedicel	F1# 194, 558, 594, 598^a^	PEDmin	3–12^b^
Long rachis	F1# 059, 405, 484, 503^a^	RLmax	3–12^b^
Short rachis	F1# 052, 241, 647, 680^a^	RLmin	3–12^b^

For phenotyping of cluster traits, samples of ripe bunches at BBCH89 were taken randomly with 3–12 replicates from replicated (*n* = 8) vines of individuals with extreme phenotype

^a^F1 individuals reported in (Richter et al. 2019) with extreme rachis or pedicel length

^b^Biological samples taken in 2013–2017 as stated in Online resource 4

### Sampling

Sampling for phenotypic evaluation: For phenotyping of PN clones at BBCH89 (ripe for harvest), ten vines per clone were chosen randomly. From each vine, a basally inserted cluster from the central shoot of the fruit cane was collected in the years 2015 and 2016 in every vineyard. A total of 16 F1 genotypes of the cross population ‘Calardis Musqué’ (GF.Ga-47-42) × ‘Villard Blanc’ with extreme rachis length and pedicel length as monitored over four years (Richter et al. 2019) were sampled with 3 to 12 biological replicates over four seasons. Bunches were cut directly at the connection with the shoot and stored at 5 °C until use.

Sampling for gene expression experiments: In the years from 2015 to 2017, the sampling time of the different ‘Pinot Noir’ clones in the three vineyard locations was fitted to hit the same developmental stage by a nonlinear cumulative degree-day (CDD)-based model (Molitor et al. 2014). The target temperature sum was 400° CDD for BBCH57 and 700° CDD for BBCH71. The CDD calculation was based on air temperatures recorded at 2 m height by the nearest weather station. Samples for gene expression analyses were collected from three randomly selected individual plants from the plantation (of about 100–200 individual plants per clone) from the lowest cluster insertion point during the developmental stages BBCH57 (just before flowering) and BBCH71 (at early fruit set) in the three years 2015, 2016 and 2017. OIV reference cultivars ‘Uva Rara’ (OIV#204 grade 1), ‘Prosecco’ (OIV#204 grade 3) and 16 F1 genotypes of the cross population ‘Calardis Musqué’ × ‘Villard Blanc’ with extreme rachis length and pedicel length were sampled with three biological replicates. Complete inflorescences were cut at the connection of peduncle and shoot and shock-frozen immediately in liquid nitrogen. A detailed schedule of the sampling and the temperature records is presented in Tables 3, 4 and Online resource 3.

**Table 3:** Sampling schedules for 12 ‘Pinot Noir’ clones spread over three locations during three seasons

Cluster type	‘Pinot Noir’ clone	Abbreviation	Palatinate		Hesse		Baden	
			BBCH		BBCH		BBCH	
			57	71	57	71	57	71
CCC	Frank Charisma	FkCH	3^a^	3^a^	3^a^	3^a^	3^a^	3^a^
CCC	Frank Classic	FkCL	3^a^	3^a^	3^a^	3^a^	–	–
CCC	Entav 777	En777	–	–	3^a^	3^a^	3^a^	3^a^
Unsteady	Geisenheim 18	Gm18	–	–	3^b^	3^b^	–	–
MBC	Geisenheim 20-13	Gm20-13	3^a^	3^a^	3^a^	3^a^	3^a^	3^a^
MBC	Freiburg 1801	Fr1801	–	–	3^b^	3^b^	3^a^	3^a^
LCC	Geisenheim 1-86	Gm1-86	3^a^	3^a^	3^a^	3^a^	–	–
LCC	Freiburg 12-L	Fr12L	–	–	3^b^	3^b^	3^b^	3^b^
LCC	Freiburg 13-L	Fr13L	–	–	3^b^	3^b^	3^b^	3^b^
LCC	Weinsberg M1	WeM1	–	–	3^b^	3^b^	–	–
LCC	Weinsberg M171	WeM171	3^a^	3^a^	–	–	–	–
LCC	Weinsberg M242	WeM242	–	–	3^b^	3^b^	–	–

For gene expression studies, samples of whole inflorescences at BBCH57 and BBCH71 were taken with three replicates from randomly selected independent vines. The expression measurements of the PN clone ‘Gm20-13’ were used for normalization of the relative PN gene expression at all three locations

– not available

^a^Three biological samples taken in 2015, 2016 and 2017

^b^Three biological samples taken in 2016 and 2017

**Table 4:** Sampling schedules for phenotypically extreme F1 individuals of the cross ‘Calardis musqué’ (formerly GF.GA-47-42) × ‘Villard Blanc’ and OIV reference varieties for loose cluster architecture

Cluster type	Variety name # F1 individual	Abbreviation	Palatinate
			BBCH71
OIV 204 reference for very loose cluster^a^	‘Uva Rara’	OIV LCC	3^b^
OIV 204 reference for loose cluster^a^	‘Prosecco’	OIV LCC	3^b^
Long pedicel^c^	F1# 212, 294, 354, 380	PEDmax	3^b^
Short pedicel^c^	F1# 194, 558, 594, 598	PEDmin	3^b^
Long rachis^c^	F1# 059, 405, 484, 503	RLmax	3^b^
Short rachis^c^	F1# 052, 241, 647, 680	RLmin	3^b^

For gene expression studies, samples of whole inflorescences at BBCH57 and BBCH71 were taken randomly with three replicates from eight cloned phenotypically extreme vines of the segregating population and three replicates of the OIV reference varieties

^a^Reference varieties for loose cluster architecture according to the OIV descriptor 204 for cluster density (OIV 2015)

^b^Three biological samples taken in 2015, 2016 and 2017

^c^F1 individuals reported in (Richter et al. 2019) with extreme measurements for rachis length and pedicel length

### Evaluation of vegetative growth

The vigor of the PN clones was determined by measuring the mass of the annual outgrowth, i.e., the weight of the ten most basally located branches on ten vines per season and location (Online resource 2, Table 5).

**Table 5:** Morphometric measurements on cluster architecture for 12 ‘Pinot Noir’ clones at BBCH89 recorded over three locations and two seasons

Trait	Abbreviation	En777	FkCH	FkCL	Fr12L	Fr13L	Fr1801	Gm20-13	Gm1-86	WeM1	WeM171	WeM242	Gm18
		Compact	Compact	Compact	Loose	Loose	Loose	Loose	Loose	Loose	Loose	Loose	Unsteady
**Berry number**	**BN** (**#**)	157.41 (± 5.83)**abc**	167.1 (± 4.82)**bc**	161.4 (± 5.93)**abc**	164.97 (± 6.17)**bc**	165.69 (± 6.05)**bc**	141.01 (± 6.13)**ab**	139.72 (± 4.53)**a**	171.7 (± 6.29)**c**	146.68 (± 7.73)**abc**	149.49 (± 8.32)**abc**	174.58 (± 12.78)**abc**	159.23 (± 11.71)**abc**
**Cluster weight**	**CW** (**g**)	181.04 (± 6.94)**bc**	211.87 (± 6.32)**bc**	193.43 (± 7.34)**cd**	248.11 (± 9.62)**de**	254.19 (± 9.63)**e**	166.76 (± 7.46)**b**	129.5 (± 4.29)**a**	246.51 (± 9.35)**de**	214.26 (± 11.71)**cde**	222.05 (± 12.57)**cde**	267.91 (± 20.62)**de**	168.84 (± 12.99)**abc**
**Mean berry Volume**	**MBV** (**cm**^**3**^)	0.84 (± 0.03)**b**	0.87 (± 0.02)**b**	0.86 (± 0.03)**b**	1.15 (± 0.03)**d**	1.06 (± 0.03)**d**	0.82 (± 0.03)**b**	0.66 (± 0.02)**a**	1.05 (± 0.03)**cd**	1.15 (± 0.04)**d**	1.12 (± 0.04)**d**	1.17 (± 0.05)**d**	0.85 (± 0.05)**abc**
Total berry Volume	TBV (cm^3^)	129.11 (± 5.35)**bc**	143.24 (± 4.62)**cd**	134.08 (± 5.5)**bcd**	190.15 (± 7.98)**f**	173.19 (± 7.09)**ef**	112.12 (± 5.42)**b**	91.1 (± 3.26)**a**	170.65 (± 7)**ef**	164.86 (± 9.74)**def**	158.3 (± 9.69)**cdef**	195.35 (± 16.25)**f**	133.53 (± 11.11)**bcde**
**Rachis length**	**RL** (**cm**)	10.91 (± 0.25)**a**	13.18 (± 0.2)**b**	12.62 (± 0.25)**b**	15.77 (± 0.26)**ef**	15.6 (± 0.25)**e**	16.26 (± 0.3)**ef**	12.83 (± 0.22)**b**	12.61 (± 0.51)**abc**	15.26 (± 0.36)**de**	15.74 (± 0.38)**def**	17.55 (± 0.51)**f**	14.39 (± 0.25)**cd**
**Shoulder length**	**SL** (**cm**)	6.93 (± 0.45)**a**	9.16 (± 0.35)**bcd**	8.01 (± 0.45)**ab**	10.34 (± 0.46)**cd**	10.17 (± 0.45)**cd**	11.04 (± 0.53)**d**	9.44 (± 0.39)**bcd**	9.11 (± 0.45)**bcd**	9.36 (± 0.65)**abcd**	9.76 (± 0.67)**bcd**	11.7 (± 0.91)**d**	7.3 (± 0.91)**abc**
**Pedicel length**	**PED** (**cm**)	0.47 (± 0.01)**a**	0.48 (± 0)**a**	0.47 (± 0.01)**a**	0.56 (± 0.01)**d**	0.56 (± 0.01)**d**	0.5 (± 0.01)**ab**	0.48 (± 0.01)**a**	0.56 (± 0.01)**d**	0.56 (± 0.01)**cd**	0.52 (± 0.01)**bc**	0.59 (± 0.01)**d**	0.5 (± 0.01)**ab**
Peduncle length	PL (cm)	1.24 (± 0.1)**abcd**	1.16 (± 0.07)**abc**	1.13 (± 0.09)**ab**	1.38 (± 0.11)**abcd**	1.58 (± 0.11)**bcd**	1.14 (± 0.11)**abc**	1.02 (± 0.08)**a**	1.72 (± 0.12)**d**	1.65 (± 0.17)**bcd**	1.42 (± 0.16)**abcd**	1.93 (± 0.27)**cd**	1.05 (± 0.19)**abcd**
Rachis weight	RW (g)	7.4 (± 0.36)**abc**	8.76 (± 0.28)**cde**	8.25 (± 0.36)**abcd**	8.94 (± 0.37)**cde**	8.21 (± 0.36)**abcd**	8.12 (± 0.42)**abcd**	6.69 (± 0.31)**a**	9.62 (± 0.36)**de**	6.78 (± 0.52)**ab**	8.72 (± 0.53)**bcde**	10.97 (± 0.73)**e**	7.81 (± 0.73)**abcde**
Rachis diameter	RD (cm)	0.4 (± 0.01)**bc**	0.35 (± 0.01)**a**	0.39 (± 0.01)**bc**	0.4 (± 0.01)**bc**	0.4 (± 0.01)**bc**	0.38 (± 0.01)**abc**	0.39 (± 0.01)**b**	0.42 (± 0.01)**c**	0.38 (± 0.01)**ab**	0.38 (± 0.01)**abc**	0.43 (± 0.02)**bc**	0.37 (± 0.02)**abc**
first internode length	L1I (cm)	1.27 (± 0.11)**a**	1.31 (± 0.09)**a**	1.26 (± 0.11)**a**	1.53 (± 0.12)**a**	1.26 (± 0.11)**a**	1.6 (± 0.13)**a**	1.3 (± 0.1)**a**	1.45 (± 0.11)**a**	1.53 (± 0.16)**a**	1.46 (± 0.17)**a**	2.08 (± 0.23)**a**	1.45 (± 0.23)**a**
second internode length	L2I (cm)	1.28 (± 0.09)**a**	1.27 (± 0.07)**a**	1.29 (± 0.09)**a**	1.49 (± 0.09)**a**	1.54 (± 0.09)**a**	1.13 (± 0.11)**a**	1.21 (± 0.08)**a**	1.37 (± 0.09)**a**	1.46 (± 0.13)**a**	1.47 (± 0.14)**a**	1.49 (± 0.19)**a**	1.35 (± 0.19)**a**
seasonal wood gain	WG (g)	790 (± 30)**bc**	716 (± 21)**abc**	613 (± 23)**a**	702 (± 27)**abc**	672 (± 25)**ab**	807 (± 36)**bc**	790 (± 26)**bc**	807 (± 31)**c**	677 (± 37)**abc**	676 (± 38)**abc**	693 (± 53)**abc**	755 (± 58)**abc**
^a^Index	BN/RL(cm)	14.39 (± 0.51)**f**	12.73 (± 0.35)**ef**	12.81 (± 0.45)**ef**	10.57 (± 0.38)**bcd**	10.77 (± 0.37)**bcd**	8.84 (± 0.36)**a**	10.94 (± 0.33)**bcd**	12.01 (± 0.42)**de**	9.8 (± 0.49)**abc**	9.48 (± 0.49)**ab**	10.16 (± 0.72)**abcde**	12.71 (± 0.7)**cdef**
^b^Index	CI_12	1.49 (± 0.07)**f**	1.19 (± 0.04)**ef**	1.17 (± 0.05)**de**	0.99 (± 0.05)**cde**	1.04 (± 0.05)**cde**	0.63 (± 0.03)**a**	0.78 (± 0.03)**b**	1.17 (± 0.05)**de**	0.93 (± 0.06)**bcd**	0.87 (± 0.06)**bc**	0.87 (± 0.08)**abcd**	1.06 (± 0.09)**bcde**
^c^Index	CI_18	4.91 (± 0.43)**f**	3.24 (± 0.22)**ef**	3.24 (± 0.28)**de**	1.77 (± 0.16)**abc**	1.96 (± 0.17)**abc**	1.31 (± 0.13)**a**	2.26 (± 0.17)**bcd**	2.31 (± 0.2)**bcde**	1.58 (± 0.2)**ab**	1.89 (± 0.24)**abc**	1.33 (± 0.23)**ab**	3.26 (± 0.57)**cdef**

Estimated (marginal) means of sub-traits and compactness indices for each clone adjusted for the effects of ‘location’ and ‘season’ as predicted from the generalized linear model ‘sub-trait’ ~ loc*year + clone (details in Online resource 6). (±) represents the standard error. Different letters indicate significantly divergent values for sub-traits and compactness indices as identified with a Tukey HSD test at significance level *α* = 0.05

^a^According to Hed et al. (2009)

^b^According to Tello and Ibáñez (2014)

^c^Based on CI-18 stated in Tello and Ibáñez (2014) but omitting seed number. Cluster architecture sub-traits indicated in bold are major contributors to cluster density levels (Richter et al. 2019)

### Phenotypic evaluation of cluster architecture sub-traits

Measurements of 12 cluster architecture sub-traits (Table 5) were used for the phenotypic assessment of the 12 PN clones. Three indices for cluster compactness were calculated. The calculation of the ratio ‘berry number/rachis length’ [BN/RL (cm), Hed et al. (2009)] and indices CI-12 [berry weight (g)]/[rachis length (cm)]^2^ and CI-18 [berry weight (g) × berry number/[peduncle length (cm) + rachis length (cm)]^2^ × rachis length (cm) × pedicel length (mm)] followed the proceedings stated in Tello and Ibáñez (2014). The 16 F1 individuals of the cross population ‘Calardis Musqué’ × ‘Villard Blanc’ were phenotypically studied for cluster architecture sub-traits during four seasons as described (Richter et al. 2019) (Online resource 4).

### RNA extraction and cDNA synthesis

For RNA extraction and cDNA synthesis, pre-bloom flowers (BBCH57) and fruit setting berries (BBCH71) were carefully removed from the inflorescence. The complete remaining stalk structure including peduncle, rachis and pedicels was ground into fine powder. All steps were performed in liquid nitrogen. Aliquots of sample tissue were mixed with 50 mg polyvinylpyrrolidone Polyclar^®^ AT (Serva Electrophoresis GmbH, Heidelberg, Germany). Total RNA extraction used the Spectrum™ Plant Total RNA Kit (Sigma Aldrich, Darmstadt, Germany), following protocol ‘A’. An on-column DNaseI digestion with RNase-Free DNase (QIAGEN, Hilden, Germany) was performed according to the manufacturer’s protocol. RNA integrity and quantity were analyzed by spectrophotometry (Clario Star 0430, BMG Labtech, Ortenberg, Germany) and checking 500 ng of total RNA by non-denaturing agarose gel (1%) electrophoresis. 250 ng of total RNA was used for first-strand cDNA synthesis with the high-capacity cDNA Transcription Kit (Applied Biosystems, Thermo Fisher Scientific, Waltham, MA, USA).

### Primer design for RT-qPCR

Primer pairs (Online resource 5) for quantitative RT-PCR (RT-qPCR) were designed as recommended in (Citri et al. 2012) using the CLC main workbench primer design software tool (CLC Main Workbench Version 8.0.1, QIAGEN www.qiagenbioinformatics.com). PCR amplification efficiencies of the primer pairs for the 91 targets and 2 endogenous control genes were validated as suggested by Schmittgen and Livak (2008). Standard RT-qPCRs were performed using the Power SYBR-Green PCR Master Mix (Applied Biosystems). The specificity of the amplification was affirmed by visual inspection of the amplification products followed by melting curve analysis and gel electrophoresis of the PCR products (after 40 thermal cycles, size inspection on 3% agarose gels).

### Expression analysis using high-throughput quantitative real-time PCR

Expression analysis applied the high-throughput BioMark™ HD (Fluidigm Corporation, Munich, Germany) system with dynamic array chips (96.96 GE IFC; Fluidigm) according to the manufacturer´s instruction. Fluorescence data recording and processing were done with the BioMark Real-Time PCR Analysis Software 3.0.2 (Fluidigm).

The overall quality score of the experiment was 0.945. Variation between the chips was low (0.92–0.97). *C*_*t*_ values of several 96.96 IFC chips were combined with their meta-data in an expression set using the R-package ‘HT-q-PCR’ (Dvinge and Bertone 2009). All *C*_*t*_ values below 5 and *C*_*t*_ values of genes showing little variation between the samples (with an inter-quartile range below 0.6) were discarded.

The relative amount of mRNA was calculated based on the *C*_*t*_ value (cycle number at threshold). The cycle threshold was determined with the automatic linear baseline setting. For normalization of the relative gene expression values, the genes *VIT_17s0000g10430* encoding glyceraldehyde-3-phosphate dehydrogenase (*GAPDH*) and *VIT_08s0040g00040* encoding ubiquitin-conjugating enzyme E2 (*UBIc*) served as references. These genes have already been successfully applied in other grapevine RT-qPCR studies, e.g., (Monteiro et al. 2013; Reid et al. 2006; Selim et al. 2012; Upadhyay et al. 2015). Their expression proved to be stable (rank invariant) in rachis tissue over clones, locations and growing seasons (as revealed with the function ‘normalizectdata’ of the package ‘HT-qPCR’). To obtain the Δ*C*_*t*_ value, the *C*_*t*_ value of each target gene was normalized by subtraction of the mean *C*_*t*_ values of the two endogenous reference genes (*GAPDH* and *UBIc*). For gene expression comparisons between F1 siblings, varieties, clones, seasons and vineyard locations, the 2^−ΔΔ*Ct*^ value was calculated (Livak and Schmittgen 2001).

### Statistics

All statistics employed R-software version 3.5.3 (R Core Team 2013). All statistic tests were set to a significance threshold of *p* = 0.05.

Cluster architecture: The environmental impact on each cluster architecture sub-trait was assessed using generalized linear models (GLM) with clone, location, season and the two-way interaction between location and season as explanatory variables. For count data, a GLM with Poisson distribution or (when overdispersed) negative binomial distribution was fitted. For strictly positive continuous responses, a Gamma-GLM with log link or a linear model was applied. Model residuals were visually assessed, and dispersion was checked when applicable. Effects were tested using type three analysis of variance and the function ‘Anova’ of the package ‘car’ (Fox and Weisberg 2011) and visualized using the function ‘alleffects’ of the package ‘effects’ (Online resource 6). Estimated marginal means, post hoc tests and pairwise comparisons with compact letter display were calculated for the effect of ‘clone’ on the response while accounting for the effects of ‘season’ and ‘location’ using the functions ‘emmeans’ and ‘CLD’ of the package ‘emmeans’ (Lenth 2019). The significance level was set to 0.05 (Table 5).

Differential gene expression, denoted as fold change (FC), was calculated using the package ‘limma’ (Matthew et al. 2015). A design matrix containing the experimental data for all investigated PN clones, varieties and F1 siblings, at up to three trial locations and three seasons, was generated with the function ‘model.matrix’. The correlation between technical replicates was estimated with the function ‘duplicatecorrelation.’ Differential gene expression was analyzed by fitting gene-wise linear models using the design matrix, the estimated correlation and the function ‘lmFit.’ To interpret different gene expression values, the empirical Bayes method was used to modify the standard errors toward a common value using the ‘eBayes’ function.

Contrast: The log_(2)_ FC (− ΔΔ*C*_*t*_) for each gene was calculated by the expression difference to the selected standard PN clone Gm20-13 using the function ‘contrasts.fit’. The relative expression (2^−Δ*Ct*^) of each Gm20-13 gene at any individual location and season of was subtracted from the (2^−Δ*Ct*^) of the test genes in all the other investigated PN clones for standardization. Following the same principle, a contrast was calculated by subtracting the (2^−Δ*Ct*^) of the genes active in compactly clustered PN clones from those in the loosely clustered varieties ‘Uva Rara’ and ‘Prosecco.’ The contrast for the F1 siblings was calculated by subtracting the (2^−Δ*Ct*^) of the test genes in F1 siblings with short pedicels and rachis lengths from the (2^−Δ*Ct*^) of the test genes in F1 individuals with extreme long rachises and pedicels, respectively. The identification of ‘regulated genes’ applied the limma package that determined differential gene expression with a threshold level of *p* ≤ 0.05.

The results of relative gene expression were displayed in heatmaps as log_2_ FC (− ΔΔ*C*_*t*_) using the package ‘pheatmap’ (Kolde 2015). Row-scaled data (gene-wise) and Euclidian distance were used for hierarchical clustering. Expression data of tested genes (log_2_ FC), displayed in box–whisker plots, were obtained in the same way as stated above, but with the contrast matrix containing additionally the biological replication (Fig. 7b, c).

Variance partition: To estimate the variation in this multilevel gene expression experiment, the package ‘variancePartition’ was used with the log_2_ of Δ*C*_*t*_. A linear mixed model with the random effects season, location, batch, biological replicate, cluster type, clone and gene pool identified the typical drivers of variance. These factors can be classified as environmental (‘season’ and ‘location’), technical (two repeated ‘batches’), biological (three independent ‘replicates’), phenotypic (‘cluster type’) and genetic (‘clone’ and ‘gene pool,’ i.e., selection background of ENTAV, Frank, Fr (Freiburg), Gm (Geisenheim) and We (Weinsberg) clones) (Hoffman and Schadt 2016).

Correlation between relative test gene expression, expressed as log_(2)_ FC (− Δ*C*_*t*_), and cluster architecture sub-trait records of PN clones for 2015 and 2016 were calculated with Spearman rank correlation test using the function ‘rcorr’ from the package ‘Hmisc’ (Harrell Jr 2015).

### Gene annotation

The gene identifiers of the Gramene database version IGGP_12x.54 (http://ensembl.gramene.org/Vitis_vinifera/Info/Index) were used to retrieve the nucleotide sequences of the candidate genes. These sequences were submitted to Blast searches (Altschul et al. 1990) in the NCBI GenBank (https://www.ncbi.nlm.nih.gov/Blast.cgi). The best match (Blastx) of the translated sequences of candidate genes with homologous genes from non *Vitis* species is used as functional annotation.

### Analysis of co-expression

An analysis of co-expression was performed with the gene expression compendium ‘Vespucci’ (Moretto et al. 2016a). The expression profiles of 14 candidate genes and *VvGRF4* were determined in 21 selected samples containing inflorescence, rachis and tendril tissue of the *V. vinifera* cultivars ‘Corvina’ and ‘Tempranillo,’ reported by Fasoli et al. (2012) and Diaz-Riquelme et al. (2014). The following ‘Vespucci’ Sample IDs have been used for co-expression analysis: ID 2210, 2211, 2225, 227, 229, 334, 335, 336, 347, 346, 348, 228, 230, 231, 232, 233, 234, 235, 307, 308 and 309. The ‘Vespucci’ inference was based on the publicly available transcriptomics data and integrated by the COLOMBOS v3.0 database (Moretto et al. 2016b).

## Results

### Trueness to type of the investigated PN clones

Microsatellite-derived markers known for their ability to reveal polymorphisms in PN clones (Pelsy et al. 2010) were applied to check the integrity of the plant material over the three plantations in Palatinate, Hesse and Baden. The data (Online resource 1) confirmed the trueness of type of the plants over all locations. The PN clones ENTAV777 and Geisenheim 1-86 showed the same genetic variation at the different locations, in agreement with the data reported by Pelsy et al. (2010).

### Cluster architecture characteristics and vitality of PN clones

**Fig. 1:**
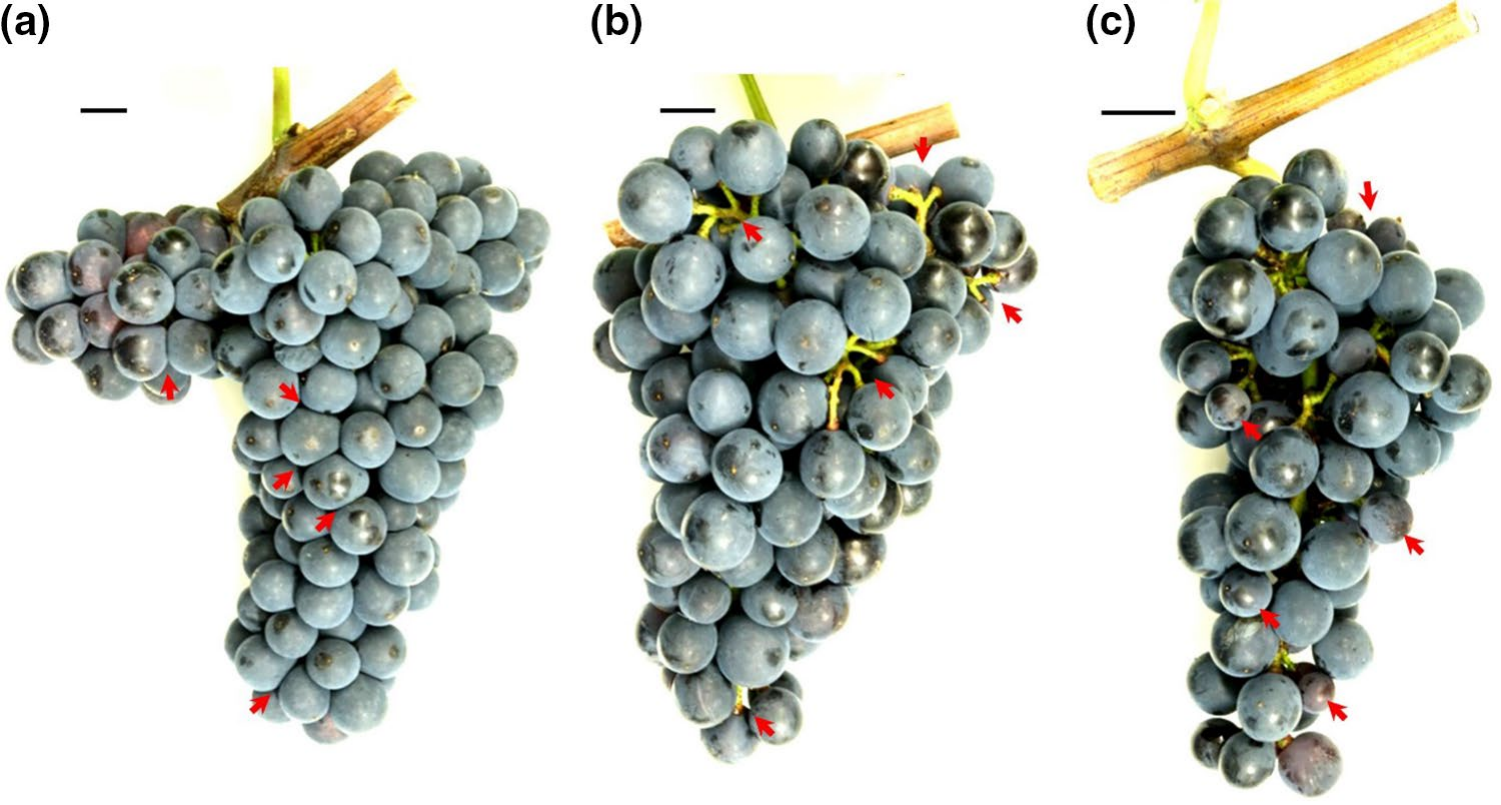
Clones of *V. vinifera* cv. ‘Pinot Noir’ with different cluster architecture. Phenological stage BBCH89 (berries ripe for harvest) was used for cluster architecture assessment. **a** The PN clone ‘Frank Charisma’ as an example for compactly clustered clones with non-circular-shaped berries due to high pressure between the berries. **b** The PN clone ‘Geisenheim 1-86’ as an example for loosely clustered clones with visibly extended pedicel length. **c** The PN clone ‘Freiburg 1801’ as an example for clones partially bearing smaller berries leading to reduced compactness (mixed berried clones). Red arrows highlight the emphasized cluster architecture feature. The size standard depicts 1 cm. Developmental stages according to Lorenz et al. (1995) (color figure online)

The typical differences in cluster architecture (CA) exhibited by PN clones at stage BBCH89 (berries ripe for harvest) are depicted in Fig. 1. The morphological characteristics of ripe bunches were evaluated in 12 PN clones spread over the three geographic locations in 2015 and 2016 at BBCH89 (Table 1, Online resource 2).

The ratio ‘berry number/rachis length’ (Hed et al. 2009) and indices CI-12 and CI-18 (Tello and Ibáñez 2014) were used to categorize the PN clones according to their cluster density. In this way, the general visual classification in loose and compact clones (Ruehl et al. 2004) was confirmed, and the clones were characterized as three CCC, two MBC and six LCC (Tables 1, 5). The clone Gm18 remained unclassified due to high variability in the measurement results recorded for the sub-traits represented in the indices.

In total, 12 sub-traits of cluster architecture (CA) were evaluated. Between the clones, 10 out of the 12 sub-traits differed significantly (The lengths of the first rachis internode (I1L) and second rachis internode (I2L) did not vary). Table 5 summarizes the morphometric data of the bunches. The loosely clustered clones from Freiburg (Fr12L, Fr13L) and from Weinsberg (WeM1, WeM171, WeM242) shared long rachis lengths and larger berry volume. The clones Fr12L, Fr13L and WeM242 showed extended pedicel lengths, as did the loosely clustered clone Gm1-86 from Geisenheim. However, the latter clone (Gm1-86) formed shorter rachises. Compact PN clones in general produced small berries with short pedicels at reduced rachis lengths. This analysis also revealed mixed berried clones that differed concerning berry volume and berry number in comparison with their co-members from the same clonal selection lines. They also exhibited a loose CA.

**Fig. 2:**
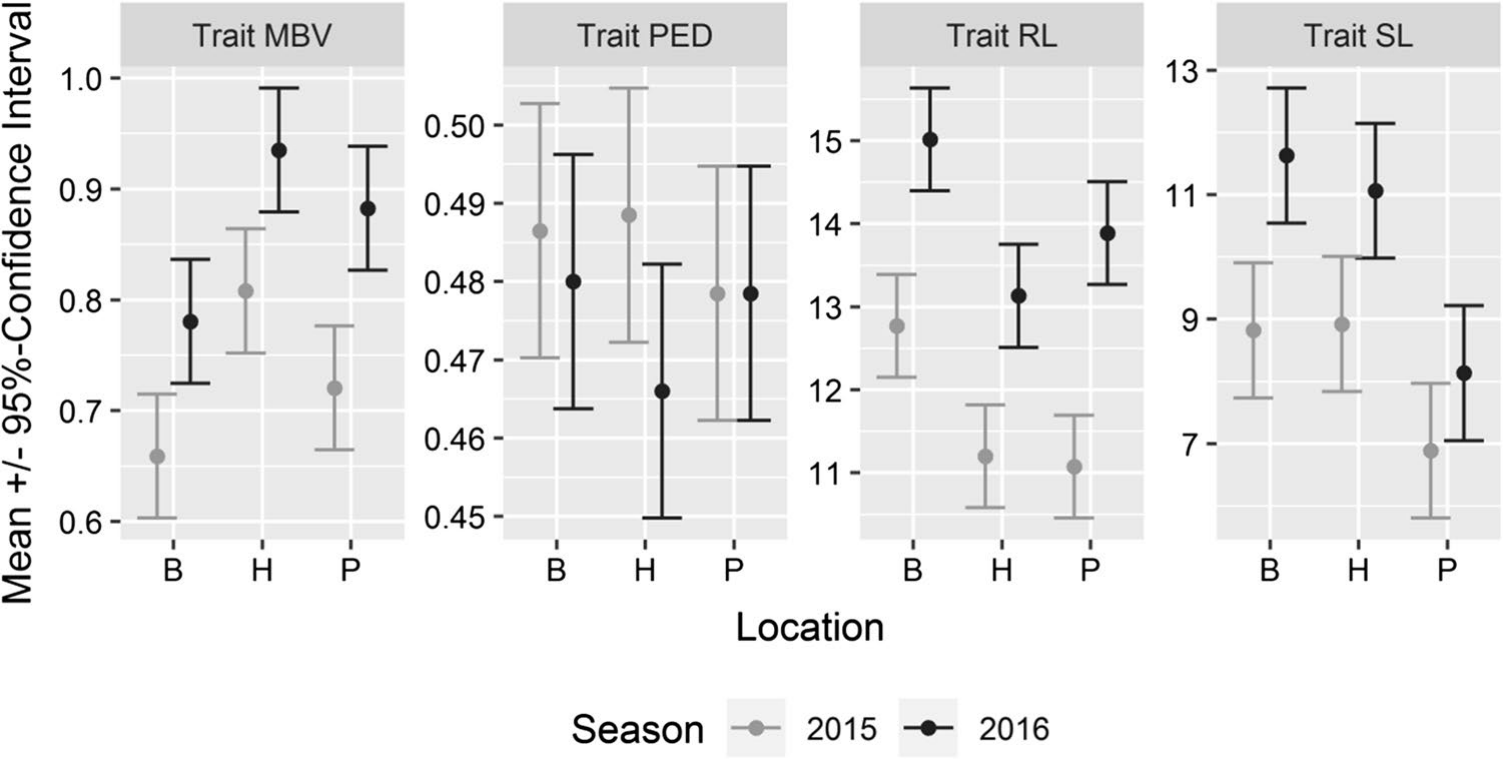
Effects of sampling locations and growing seasons on cluster architecture sub-traits for the ‘Pinot Noir’ clones Gm20-13 and FkCH. These two clones could be sampled across all seasons and locations (*n* = 120). Estimated marginal means and 95% confidence intervals were obtained from generalized linear models. The CA sub-traits rachis length (RL), shoulder length (SL) and mean berry volume (MBV) were clearly influenced by ‘season.’ In contrast, pedicel length (PED) was affected neither by ‘season’ nor by ‘location’ (Online resource 6)

The effects of the environmental factors ‘season’ and ‘location’ on CA were evaluated using the clones Gm20-13 and FkCH since these clones were common to all three locations (Hesse, Baden and Palatinate). The evaluation of generalized linear models revealed that ‘season’ affected berry number (BN), mean berry volume (MBV), total berry volume (TBV), rachis length (RL), shoulder length (SL) and rachis weight (RW). The factor ‘location’ influenced cluster weight (CW), mean berry volume (MBV), total berry volume (TBV), rachis length (RL), shoulder length (SL) and rachis weight (RW). The values for peduncle lengths (PL) and pedicel lengths (PED) in Gm20-13 and FkCH were stable and did not differ between locations and seasons (Fig. 2, Online resource 6a and 6b).

In addition to CA sub-traits, the annual wood gain was recorded as indicator of plant vigor (Table 5). The values of clones Gm20-13 and FkCH attained during the seasons 2015 and 2016 differed significantly between the three locations (Online resource 2). The highest wood gain per vine was achieved in Baden (average 1136 g, integrated management), followed by Hesse (average 758 g, integrated management) and Palatinate (average 456 g, vineyard under organic management). Wood gain (WG) was not significantly affected by season (Online resource 6). The morphometric measurements served to study differential gene expression in association with cluster architecture features.

### Identification of genes regulated in association with cluster architecture sub-traits

In total, 80 candidate genes were selected based on a previous RNA-Seq study reported by analysis of each two loosely and compactly clustered PN clones (Rossmann et al. 2019). These genes had shown a significant fold change of at least 1.5 between loose and compact clones. In addition, 11 candidate genes were selected for analysis based on their implication in inflorescence development as reported in the literature. A list of all genes is presented in Online resource 5. The gene *VvGRF4* was included to check its implication in cluster compactness in an extended set of ‘quasi isogenic’ PN clones from various selection backgrounds and over multiple environments.

**Fig. 3:**
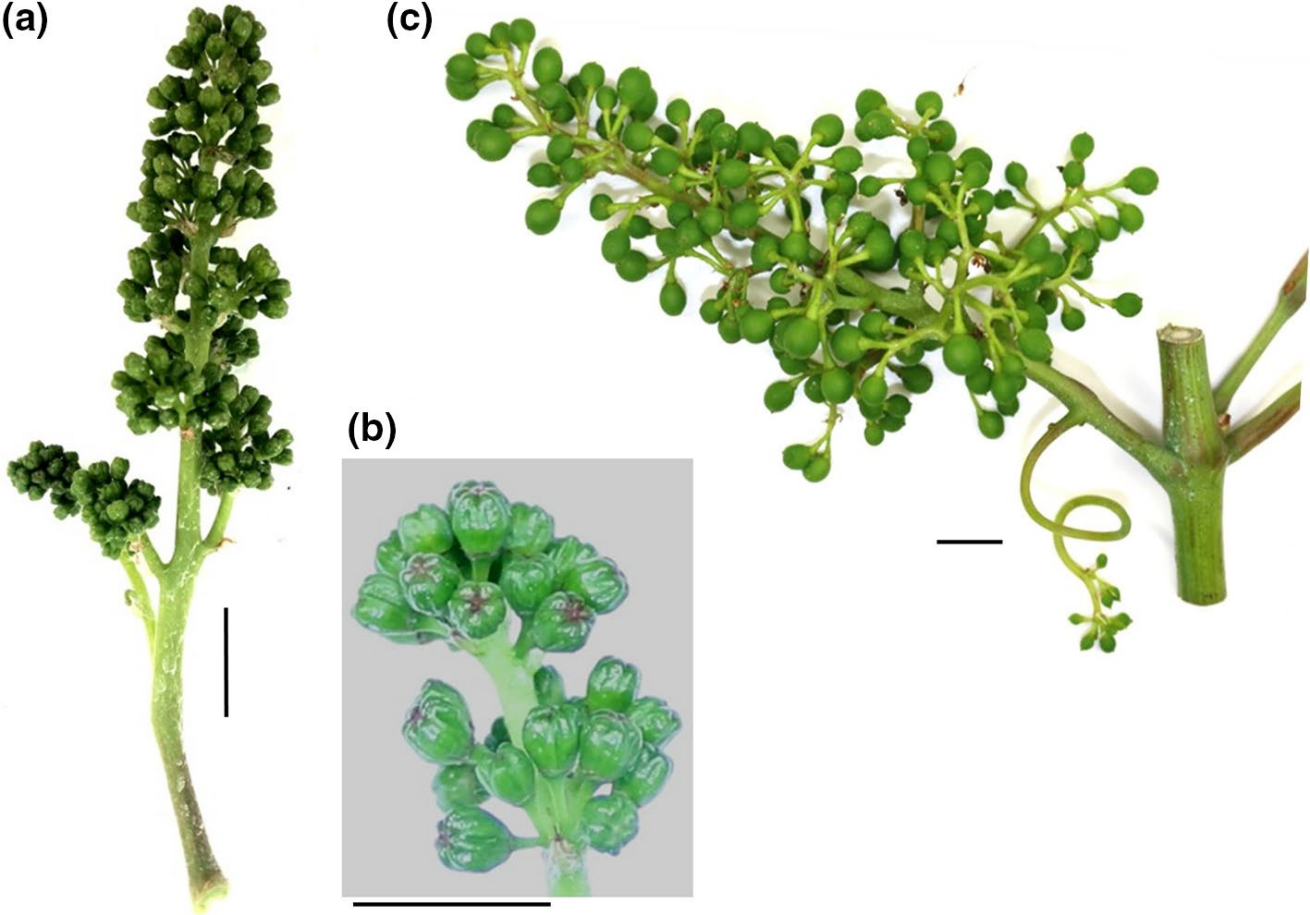
For differential gene expression studies, BBCH57 (**a**) (just before flowering with still closed flower caps (**b**)] and BBCH71 (**c**) (berry set) samples were used. For each time point, three biological replicates were collected from different vines. The sampled vines were chosen randomly within a plantation of several hundred individuals of each clonal variant. Only vines without any indication of pathogen infection or physiological disorder were sampled

Accelerated inflorescence growth of loosely as compared to compactly clustered PN clones just before flowering (BBCH57) and at early fruit set (BBCH71) has been reported (Richter et al. 2017). Hence, these time points were chosen for the expression analysis in the 11 PN clones of LCC, MBC and CCC phenotype (Fig. 3). The clone Gm20-13 had a special distinct phenotype (small berries, short rachises) and served as reference to standardize the gene expression data.

**Fig. 4:**
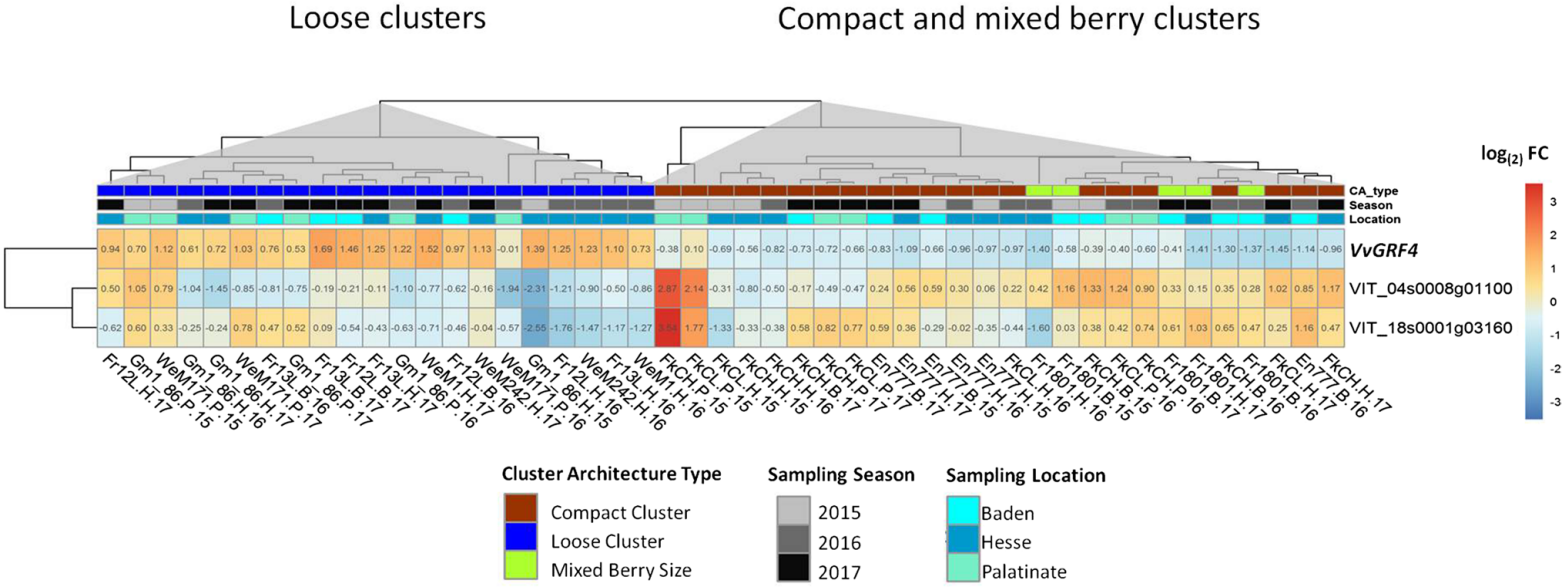
Heatmap of the averaged (three biological and two technical replicates) relative gene expression values as log_(2)_ FC (− ΔΔ*C*_*t*_) of selected genes at BBCH57. The gene expression relative to the mean of *GAPDH* and *UBIc* was analyzed just before flowering (BBCH57) and standardized relative to the PN clone Gm20-13. The rows show the relative expression of the genes. The columns represent the ‘Pinot Noir’ samples. The clones are indicated at the bottom with their abbreviated name, their location (*B* = Baden, *H* = Hesse, *P* = Palatinate) and the year of sampling (15 = 2015, 16 = 2016, 17 = 2017). Hierarchical clustering (based on Euclidian distances) revealed similarities in gene regulation in the PN clones depending on their cluster architecture (CA) type. LCCs are separated from CCCs and MBCs

Quantitative real-time PCR was performed on developed inflorescences (BBCH57) and on young clusters at fruit set (BBCH71). Data were normalized to the internal controls (*GAPDH* and *UBIc*), standardized with Gm20-13 values and reported as logarithm of the fold change (− ΔΔ*C*_*t*_). In total, 40 genes at BBCH57 and 81 genes at BBCH71 appeared differentially expressed between the PN clones of LCC, MBC or CCC phenotype (Online resource 7). Out of these, 15 genes were differentially expressed over all conditions, independently from environmental factors ‘season’ and ‘location’ (as inferred with moderated T-statistics using empirical Bayesian modeling, Smyth 2004). Three genes were consistently differentially active at the early stage of BBCH57 (Fig. 4). They included the gene encoding transcription factor VvGRF4, as expected from the former study (Rossmann et al. 2019), assessed here in a larger clone set. In addition, the two genes *VIT_04s0008g01100* (encoding a cytochrome P450 CYP711A1-like gene, named *MAX1* in Arabidopsis) and *VIT_18s0001g03160* (annotated as a WAT1-related protein) were differentially expressed at this early stage under all conditions.

**Fig. 5:**
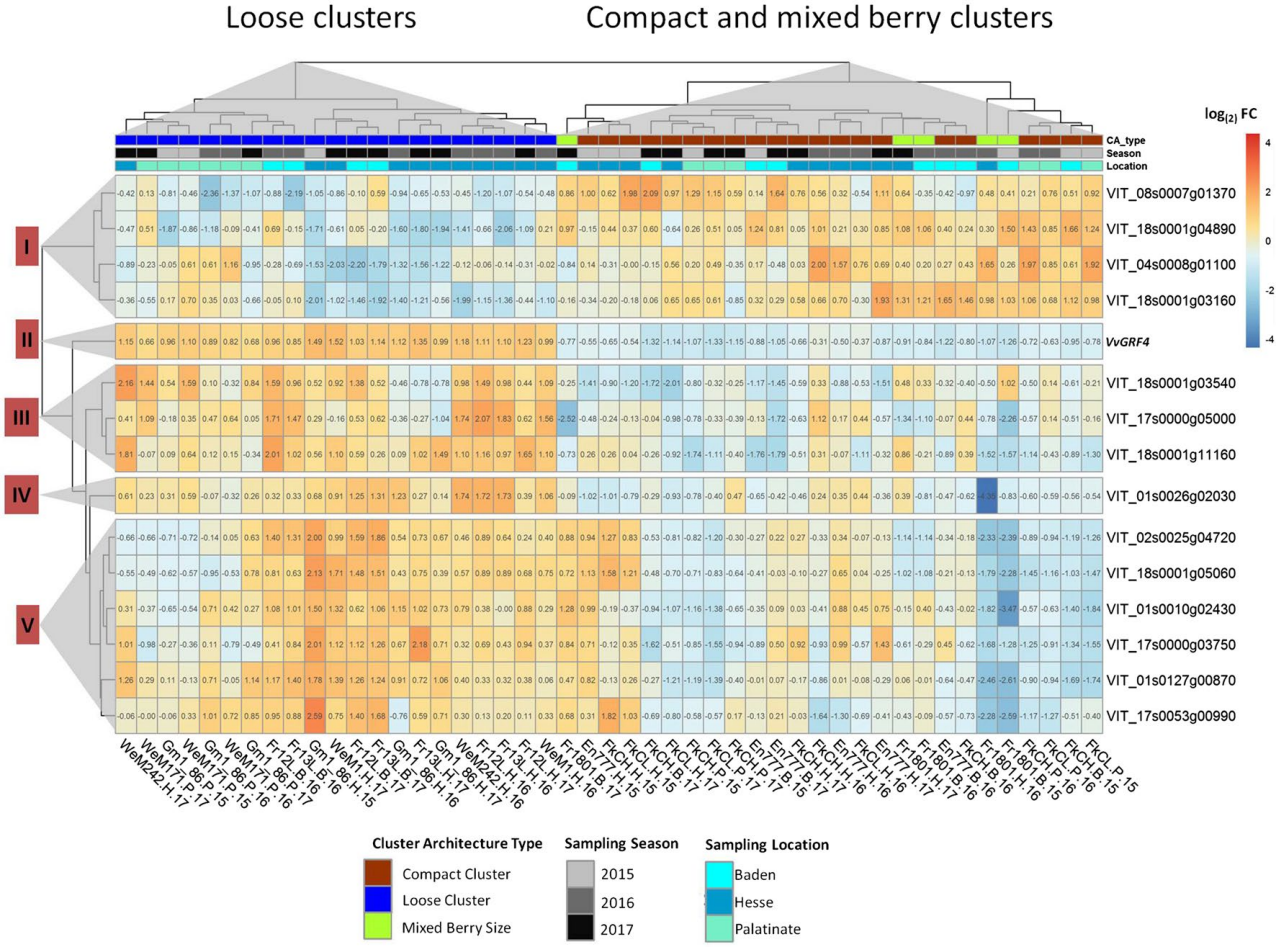
Heatmap of the averaged (three biological and two technical replicates) relative gene expression values as log_(2)_ FC (− ΔΔ*C*_*t*_) of selected genes at BBCH71. The gene expression relative to the mean of *GAPDH* and *UBIc* was analyzed just after flowering (BBCH71) and standardized relative to the PN clone Gm20-13. The rows show the relative expression of the genes. The columns represent the ‘Pinot Noir’ samples. The clones are indicated at the bottom with their abbreviated name, their location (*B* = Baden, *H* = Hesse, *P* = Palatinate) and the year of sampling (15 = 2015, 16 = 2016, 17 = 2017). Hierarchical clustering (based on Euclidian distances) revealed similarities in gene regulation in the PN clones depending on their cluster architecture (CA) type. LCCs are separated from CCCs and MBCs. The genes expression data form five clusters of similar patterns (as indicated by numbers at the left-hand side)

*VvGRF4* was differentially expressed both at BBCH57 and at BBCH71. In agreement with former results, its activity was high in LCC clones and down-regulated in CCC (Figs. 4, 5). The expression of *VvGRF4* in MBCs resembled the pattern seen in CCCs.

After fruit set and begin of fruit development (BBCH71), 11 more genes were found to be differentially expressed between loose and compact PN clones independently from all seasons and locations.

Hierarchical clustering based on their expression values grouped them into five clusters of similar expression patterns (Table 6, Fig. 5). Clustering of PN clones showed a clear separation of LCCs from CCCs and MBCs (Fig. 5).

**Table 6:**
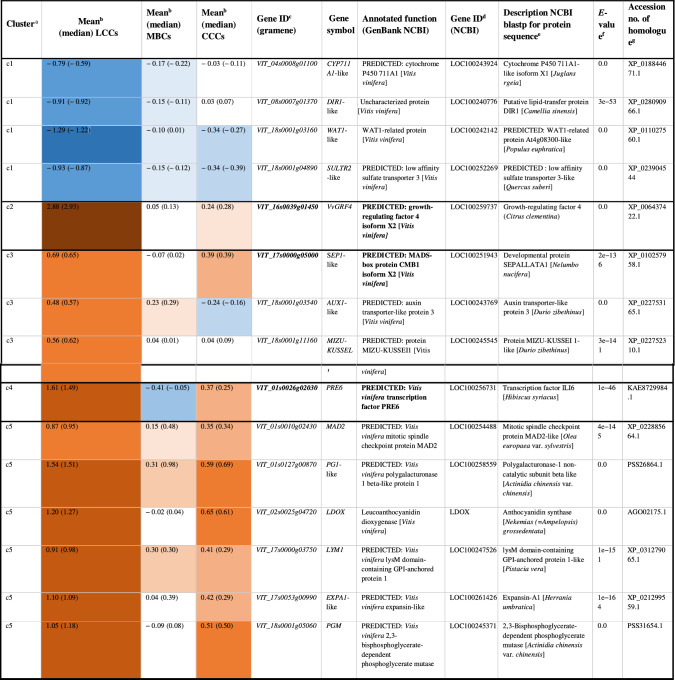
Average gene expression fold change log_(2)_ FC (− ΔΔC_*t*_) at early fruit development stage (BBCH71) in loosely clustered clones (LCCs), mixed berried clones (MBCs) and compactly clustered clones (CCCs) as compared to the standard ‘Pinot Noir’ clone Gm20-*13

(a) Hierarchical clusters (Euclidian distances) of the relative gene expression (Figs. 4, 5) (b) Clone group specific mean and median values of relative expression. The color code corresponds to the colors used in the heatmap in Figs. 4 and 5 and indicates changes based on the mean expression value. (c) Identifier from the Gramene data base (http://ensembl.gramene.org/Vitis_vinifera/) and functional annotation of the genes at NCBI Genbank (https://www.ncbi.nlm.nih.gov/nuccore) (d) Gene identifier from NCBI (e) Best match (Blastp) of the translated amplified sequences of candidate genes with homologous genes from non Vitis species (https://blast.ncbi.nlm.nih.gov/Blast.cgi) (f) Quality estimator value for similarity between sequences (g) Accession number of homologous genes in the NCBI database

In expression cluster I, the transport- and phytohormone-related genes *VIT_04s0008g01100* (*CYP711A1*-like), *VIT_08s0007g01370* (*DIR1*-like), *VIT_18s0001g03160* (*WAT1*-like) and *VIT_18s0001g0489* (*SULTRA3*-like) were down-regulated in the majority of LCCs, while they showed only little expression changes in most MBCs and CCCs. The gene *VvGRF4* formed a separate cluster II and followed a homogenous differential expression pattern specific to loose and compact/mixed berried clones, respectively. It was more active in LCC clones. Cluster III combined the genes *VIT_17s0000g05000* (S*EP1*-like), *VIT_18s0001g03540* (*AUX1*-like) and *VIT_18s0001g11160* (*MIZU*-*KUSSEL1*-like). The products of these genes relate to transcription regulation (transcription factor SEPALLATA1-like), auxin transport and auxin homeostasis. They were up-regulated in most LCCs to a much larger extent than in CCCs. Cluster IV contains gene *VIT_01s0026g02030*. It probably encodes a non-DNA binding basic helix-loop-helix (bHLH) transcription factor PRE6. For this transcription factor gene, the LCCs showed higher expression than the CCCs. The MBCs showed a heterogeneous range of differential expression extending from − 4.35 to 0.39. In cluster V, expression patterns showed the highest heterogeneity. The genes *VIT_01s0010g02430* (*MAD2*-like), *VIT_01s0127g00870* (*PG1*-like), *VIT_17s0000g03750* (*LYM1*) and *VIT_17s0053g00990* (*EXPA1*-like) encode proteins related to cell wall synthesis or cellular growth. The products of the genes *VIT_02s0025g04720* (*LDOX*) and *VIT_18s0001g05060 (PGM)* are associated with pro-anthocyanidin synthesis resp. glycolysis/gluconeogenesis. Few CCC samples showed divergent (up-regulated) gene expression affected by ‘season’ and ‘location’ (e.g., Hesse 2015). Interestingly, the LCC samples from Palatinate (under organic farming) showed repression for four genes in cluster V in contrast to the clones from the other locations managed by integrated viticulture practices (Fig. 5). The expression changes are summarized in Table 6.

### Variance of gene expression in PN explained by experimental factors

**Fig. 6:**
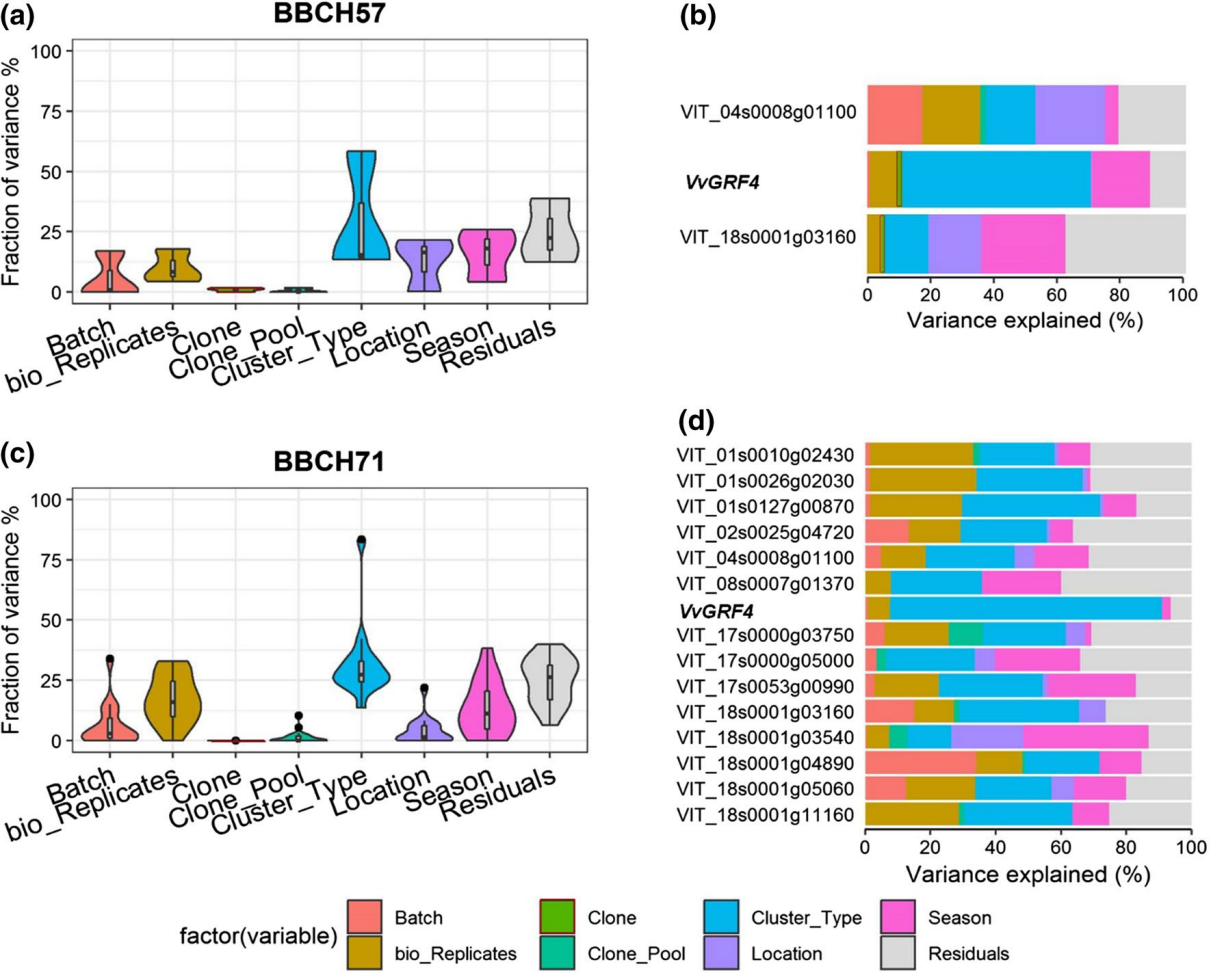
Variance partition analysis using experimental factors to assess the percentage of the explained variance of gene expression. The violin plots (**a**, **c**) indicate the explained variances in overall gene expression values log_(2)_ (Δ*C*_*t*_) on the *y*-axis, while the *x*-axis depicts the factors of variance: cluster type (loose, mixed berried, compact), bio-replicates, (biological replicates, *n* = 3), season, batch (technical replicates, *n* = 2), location, gene pool (selection background), clone (11 ‘Pinot Noir’ clones) and the residuals. The bar plots (**b**, **d**) depict the amount of variance explained by each factor on the individual gene’s expression

In order to determine to which extent the modulations of gene expression were affected by the experimental factors, a variance partition analysis was carried out. For all the identified genes, the factor ‘cluster type’ explained a substantial percentage of the variance in gene expression. The factors ‘location’ and ‘season’ also showed clear effects (Fig. 6, Online resource 8).

At the early time point, (BBCH57) the main cause of variance for *VvGRF4* was ‘cluster type’ (58% explained variance). For *VIT_18s0001g03160* (a vacuolar auxin transporter, *WAT1*-*like*), it was ‘season’ (26%). The variance of *VIT_04s0008g01100* (*CYP711A1*-like) was mainly explained by the factor ‘location’ (22%) at this early developmental stage.

At the later developmental stage, BBCH71, the factor ‘cluster type’ was the major determinant of gene expression variation of almost all 15 investigated genes. The sole exception was *VIT_18s0001g03540* (*AUX1*-*like*, with only 14% of variance explained by ‘cluster type’ but over 20% by the factor ‘location’). The variance of *VvGRF4* gene expression was explained to more than 80% by ‘cluster type,’ and the environment caused little variation (‘location’ 0%, ‘season’ 2.6%). The factor ‘season’ was an important determinant of gene expression variation explaining more than 20% of variance for the genes *VIT_08s0007g01370* (*DIR1*-*like*), *VIT_17s0000g05000* (*SEP1*-*like*), *VIT_17s0053g00990* (*EXPA1*-*like*) and *VIT_18s0001g03540* (*AUX1*-*like*) (Fig. 6, Online resource 8).

The gene *VIT_18s0001g04890* (*SULTR2*-*like*) was affected by factor ‘batch’ (technical replicates), and the genes *VIT_01s0010g02430* (*MAD2*)*, VIT_01s0026g02030* (*PRE6*)*, VIT_01s0127g00870* (*PG1*-*like*) and *VIT_18s0001g11160* (*Mizu*-*Kussel1*-*like*) varied to some extent also over the biological replicates (Online resource 8).

### Correlation of gene expression with sub-traits of cluster architecture and wood gain

At the early stage of BBCH57, the relative expression of *VvGRF4* (log_(2)_ FC) was strongly correlated with the sub-traits mean berry volume (MBV; *r* = 0.87/0.90) and pedicel length (PED; *r* = 0.92/0.89) in both years. In contrast, the activity of genes *VIT_04s0008g01100* and *VIT_18s0001g03160* correlated inversely with MBV and PED (Table 7). At this time, there was no significant correlation to shoulder length (SL).

**Table 7:**
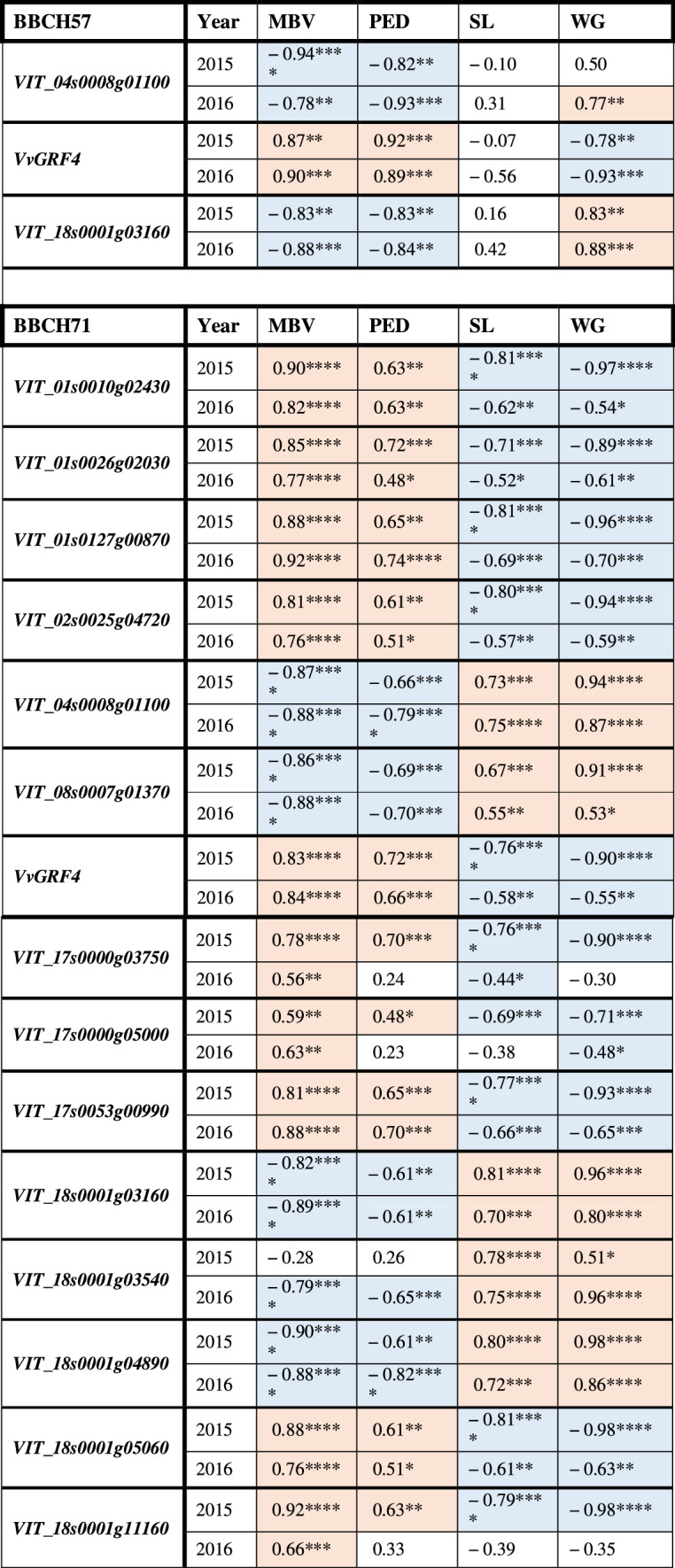
Coefficient of correlation (*r*) between the relative expression changes of selected genes and key sub-traits of cluster architecture and wood gain (for abbreviations see Table 5)

The gene expression relative to *GAPDH* and *UBIc* (log_(2)_FC) was measured just before flowering (BBCH57) and just after flowering (BBCH71). The results for cluster architecture sub-traits of ‘Pinot Noir’ clones were recorded at ripe grape clusters stage BBCH89. Wood gain was recorded after leaves had fallen (BBCH97)

Spearman correlation (*r*) is significant with *p < 0.05, **p < 0.01, ***p < 0.001 and ****p < 0.0001

Positive correlation is highlighted in light red, negative correlation in light blue

During 2015 and 2016, at developmental stage BBCH71, all selected genes changed expression correlated with at least one of the sub-traits mean berry volume (MBV), pedicel length (PED) or shoulder length (SL) (Table 7). Three main trends appeared in both seasons. I) 11 genes with significant correlation with MBV also correlated with PED in the same sense (positive or negative correlation). Genes with correlation with SL often co-correlated with plant vigor (measured as wood gain, WG). II) The correlations with MBV/PED in general appeared inverse to the correlations observed to SL/WG (Table 7, Online resource 9). III). None of the 15 genes showed any significant correlation with the sub-traits berry number (BN), cluster weight (CW) or rachis length (RL) (Online resource 9).

Interestingly, at BBCH71 the correlation of the genes expression with MBV was generally stronger than to PED. All genes showed regulation correlated with the sub-trait shoulder length (SL) in at least one season.

### Correlation in between the modulated genes

In general, the correlation among the differentially expressed genes was strong, with the sole exception of *VIT_18s0001g03540* (Online resource 9).

Consistent with the gene expression clusters (Fig. 5), the genes that were positively correlated with MBV and PED also correlated positively with the genes of the expression clusters II to V, but negatively with the genes of cluster I. On the contrary, the genes that correlated negatively with MBV and PED also correlated negatively with all genes in expression clusters II to V, but positively with the genes in cluster I (Online resource 9).

The three genes *VIT_01s0026g02030 (PRE6)*, *VvGRF4* and *VIT_17s0000g05000* (*SEP1*-like) encode putative transcription factors. At BBCH57, the expression of *VvGRF4* correlated negatively with the genes differentially expressed at this developmental stage. This negative correlation continued to the later stage. At BBCH71, the expression of the ten other regulated genes was always correlated with the transcriptional activity of the three transcription factor genes in the same sense (with the sole exception of the gene *VIT_18s0001g04890* that correlated with *VIT_17s0000g05000* only during the season of 2015) (Table 8). The three transcription factor genes correlated positively with each other.

**Table 8:**
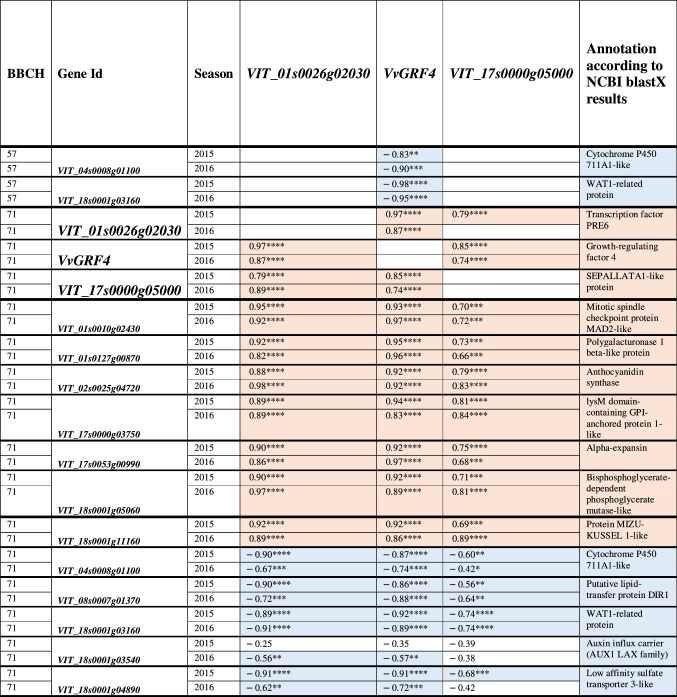
Coefficient of correlation for relative gene expression (log_(2)_FC) between the three putative transcription factors and differentially regulated genes

Spearman correlation (*r*) is significant with *p < 0.05, **p < 0.01, ***p < 0.001 and ****p < 0.0001

Positive correlation is highlighted in magenta, negative correlation in light blue

### Expression of cluster architecture-associated genes in alternative genetic backgrounds

**Fig. 7:**
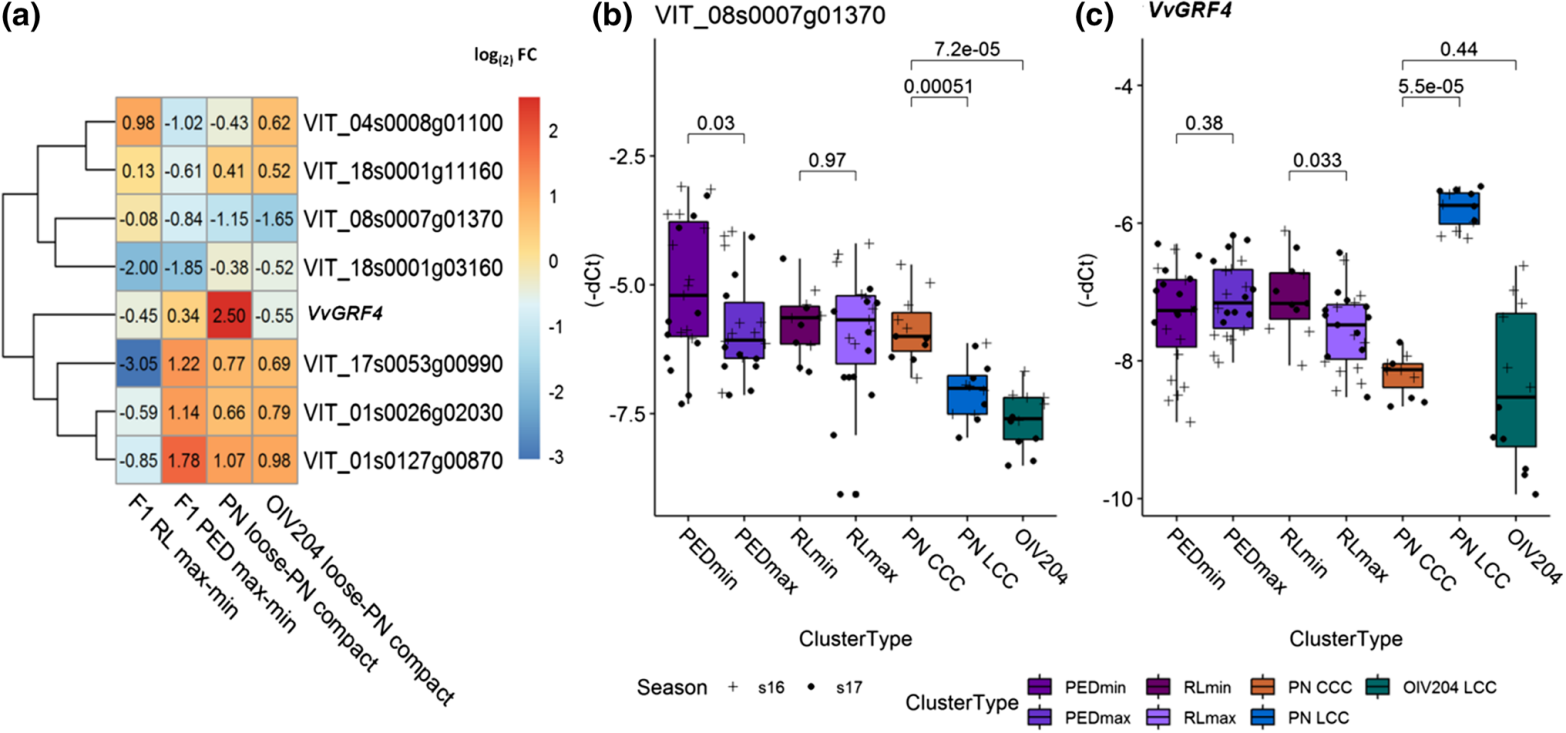
Differential expression of CA-related genes identified in PN in genetically distant backgrounds. Values from PN clones are included for comparison. **a** Heatmap of the averaged relative gene expression values as log_(2)_ FC (− ΔΔC_*t*_) at BBCH71 (just after flowering). The gene expression relative to the mean of *GAPDH* and *UBI*c was analyzed in three biological replicates. For gene activity in F1 individuals, a contrast to the mean of four individuals with short pedicels and short rachis was used, respectively. For standardization of loosely clustered individuals of OIV reference varieties, a contrast to the two compactly clustered PN clones, Frank Classic and Frank Charisma, was calculated. **b**, **c** Fold change (− ΔC_*t*_) of *VIT_08s0007g01370* (**b**) and *VvGRF4* (**c**) relative to the internal control genes during two seasons at BBCH71 as measured in phenotypic and genotypic diverse individuals grouped according to their cluster architecture type. Cluster architecture types consist of the following individuals: PEDmin and PEDmax, four F1 hybrids each were grouped according to pedicel length. RLmin and RLmax, four F1 hybrids each were grouped according to rachis length. PN LCC, loosely clustered ‘Pinot Noir’ clones Gm1-86 and WeM171. PN CCC, compactly clustered ‘Pinot Noir’ clones Frank Classic and Frank Charisma. OIV 204, ‘Uva Rara’ and ‘Prosecco,’ two OIV reference varieties of cluster density OIV descriptor#204 for loose cluster architecture. Indicated *p* values were generated with Wilcoxson’s test between group means of cluster architecture types

The differential gene expression of the 15 genes identified in the PN clones was tested for maintenance of their association with the sub-traits of CA in completely different genetic backgrounds. To this purpose, the OIV reference varieties for loose cluster architecture ‘Uva Rara’ and ‘Prosecco’ were analyzed. In addition, 16 interspecific F1 hybrids from a cross population of ‘Calardis Musqué’ (formerly GF.GA-47-42) × ‘Villard Blanc’ (Zyprian et al. 2016) were chosen for this broadened analysis. These samples comprised four genotypes each showing maximal or minimal pedicel lengths and each four individuals of maximal or minimal rachis lengths as characterized in Richter et al. (2019) and detailed (including *T* Test) in Online resource 4. They were included in the high-throughput RT-qPCR chips at stage BBCH71. Out of the 15 genes with differential expression between loose and compact quasi-isogenic PN clones, seven genes maintained their differential expression in individuals of contrasting cluster architecture sub-traits in this diverse genetic background (Fig. 7a, Online resources 10 and 11).

The gene encoding VvGRF4 lost its association with CA within these genetically different grapevine samples (Fig. 7a, c). Its differential expression was restricted to the PN clones. It was neither regulated in the OIV reference varieties ‘Uva Rara’ and ‘Prosecco’ nor the F1 hybrids of the cross population. Although the investigated F1 siblings exhibited extreme pedicel lengths difference, and pedicel length is a discriminant between loose and compact PN clones, no significant correlation of *VvGRF4* gene expression modulation in relation to pedicel lengths was identified (Fig. 7c).

Particularly, the three genes *VIT_01s0026g02030 (PRE6), VIT_01s0127g00870* (*PG1*-like) and *VIT_17s0053g00990* (*EXPA1*-like) genes were significantly up-regulated (FC ~ 1.6–2.1) in the OIV reference varieties for loose cluster architecture ‘Uva Rara’ and ‘Prosecco’ (related to compact PN clones, Fig. 7a).

The gene *VIT_08s0007g01370* (*DIR1*-like), which showed down-regulation in loose PN clones, was also expressed at considerably reduced level in the loose OIV reference varieties (Fig. 7a, b).

Regarding the F1 siblings with long rachises, the three genes *VIT_01s0026g02030 (PRE6), VIT_01s0127g00870* (*PG1*-*like*, *jp650*-like) and *VIT_17s0053g00990* (*EXPA1*-like) showed reduced expression as compared to siblings with short rachis length. In contrast, F1 siblings with long pedicels showed higher expressions of these genes in comparison with their siblings with short pedicels (Fig. 7a, Online resource 10).

The expression of *VIT_18s0001g03160* (*WAT1*-like) appeared 3.6–4-fold down-regulated in F1 hybrids with long pedicels and large rachis length. The F1 genotypes #484 and #503 appeared particularly diminished for expression of *VIT_18s0001g03160* and likewise for the gene *VIT_17s0053g00990*.

The genes *VIT_04s0008g01100* (*CYP711A1*-like) and *VIT_18s0001g11160* (*MIZU*-*KUSSEL1*-like) showed a contrasting regulation pattern regarding the four experimental sets (Fig. 7a). The loosely clustered OIV#204 reference varieties and F1 hybrids with long rachis were more actively expressing these genes, while F1 hybrids with long pedicels were found reduced in the activity of these two genes.

### Co-expression network analysis

To learn more about the regulatory networks involved in cluster morphogenesis, the gene expression data obtained in this study were checked for co-expression within other publicly available grapevine transcriptomic datasets. The co-expression network, calculated with the grapevine gene expression compendium ‘Vespucci’ (Moretto et al. 2016a), revealed that 11 of the 15 genes are part of a co-expression network when examined within the expression data of ‘Corvina’ (Fasoli et al. 2012) and ‘Tempranillo’ (Diaz-Riquelme et al. 2014) samples. The genes within the network had manually annotated functions comprising auxin signaling, auxin transport, cell cycle and flower development. The genes *VIT_04s0008g01100* (*CYP711A1*-*like*), *VIT_08s0007g01370* (*DIR1*-*like*), *VIT17s0000g05000* (*SEP1*-*like*) and *VIT_18s0001g05060* (*PGM*) do not belong to any co-expression network (Diaz-Riquelme et al. 2014, Fasoli et al. 2012) represented in the available data sets.

## Discussion

This study analyzed 92 genes involved in the determination of loose cluster architecture in different PN clones. The implication of *VvGRF4*, recently identified as an important regulator of cluster architecture in four PN clones (Rossmann et al. 2019), was confirmed here in a wider genetic range of PN. Seven of these genes could be validated for their association with cluster architecture in completely different genetic background, in OIV reference varieties for loose cluster architecture and in phenotypically extreme F1 siblings from a controlled cross. These included the gene annotated as encoding transcription factor PRE6. The regulation of *VvGRF4*, in contrast, was limited to the PN clones of selection lines with different pedicel length. Such restriction of intravarietal variance was also reported in Fernandez et al. (2010, 2014). The authors detected a mutation causing alterations of inflorescence morphology in the promoter of *VvTFL1A* in somatic variants of the cultivar ‘Carignan.’ However, the authors could not find that specific mutation in a population of 140 varieties with diverse cluster architecture.

The phenotype of an organism is determined by a combination of its genotype (*G*), the environment (*E*) and their interaction (*G* × *E*) (Grishkevich and Yanai 2013). Considering this fact, it is desirable to dispose high numbers of clonal individuals spread over several locations for investigation. However, for perennial crops like grapevine, this requirement is difficult to fulfill. Establishment of controlled vineyards raised from certified plant material with ample material to allow random sampling is time-consuming and expensive. The PN clones in this study needed to be grown in homogeneous plots and grafted on the same rootstock cultivar to avoid transcriptomic shifts in the scion and influences on yield and vigor by the rootstock (Chitarra et al. 2017). The experimentation here was therefore restricted to clonal material available at the collaborating nurseries and the cultivar repository at the JKI. The three plantations were under different viticulture systems with organic viticulture at Geilweilerhof and integrated management at the nurseries. This fact should delimit the identification of genetic components affecting the phenotype of cluster architecture to those that operate autonomously from environmental conditions.

Organic or integrated vineyard management may influence CA development. Döring et al. (2015) used ‘Riesling’ vines (on rootstocks ‘Börner’ and ‘SO4’) to compare growth and yield parameters in relation to viticulture systems of integrated and organic production. The authors reported significant lower cluster and berry weight under organic management. The latter parameter (berry weight) could be regarded as equivalent to mean berry volume (MBV) analyzed in this study. Interestingly, in the study here, the vineyard in Baden (integrated) had lower MBV as compared to the organically maintained field in Palatinate. It might be possible that there is a difference in grapevine cultivars regarding their requirements for nutrients and a cultivar-specific shift to promote generative development under nutrient shortage. This may be indicated by the lower wood gain observed in the organically managed vineyard.

In total, 12 different PN clones of various cluster architecture types were characterized for cluster sub-traits. Ripe bunches were measured for two seasons in three different environments. Enlarging the range of CA types investigated previously (conducted on two loose and two compact PN clones), the additional cluster type of ‘mixed berried-clones’ was included newly in this investigation. These MBC clones result in rather loose bunches at ripeness, due to the presence of interspersed small berries within the clusters. Among the cluster architecture characteristics studied over all clones, the sub-traits MBV (mean berry volume), RL (rachis length) and PED (pedicel lengths) emerged as the most relevant determinants of overall cluster architecture. This finding is in agreement with the results from the former genetic study on QTLs related to cluster architecture mapped on a segregating population independent from the PN gene pool (Richter et al. 2019). Particularly, the sub-trait PED (pedicel length) was clearly discriminant between compact and loosely clustered PN clones (Table 5). Formation of the pedicel is largely influenced by cell number, and the long pedicels possess a higher number of cells in comparison with short pedicels of compact bunches in PN (Rossmann et al. 2019). This phenomenon is linked to the differential gene regulation of *VvGRF4* due to its mutation in the microRNA binding site. In this case, there appears to be an obvious direct influence of the genetic constitution, specific for ‘Pinots.’ Quite in contrast, the phenotypically extreme F1 siblings concerning pedicel length were differentially regulated in the activity of transcription factor gene *PRE6*, but not in *VvGRF4* expression (Fig. 7a, c). The gene encoding *PRE6* is enclosed in the confidence interval of a QTL for pedicel length and cluster architecture scored according to OIV descriptor #204 identified in the former genetic study (Richter et al. 2019). These findings may allow us to conclude that specifically the sub-trait pedicel length is primarily controlled by the genetic constitution and less affected by environmental effects. This finding is of high relevance for promising application in grapevine breeding and the development of genetic markers.

Genetic components affecting mean berry volume (MBV) are also operating, since many genes differentially expressed in association with this sub-trait were identified. In the PN samples, essentially all of the 15 generally CA-associated genes correlated with MBV (Table 7). The sub-trait rachis length (RL) turned out as relevant characteristic of overall cluster architecture, but did not show any significant correlation with the genes investigated.

The developmental period from pre-anthesis to beginning berry formation was chosen to study gene regulation as the stage relevant for the constitution of final cluster compactness (Tello and Forneck 2018). This period was reported to be important for the modulation of cluster architecture sub-traits berry number (Bessis and Fournioux 1992), rachis length (Shavrukov et al. 2004) and berry volume (Houel et al. 2013). Particularly, the latter traits constitute loose or compact CA in a cultivar-dependent manner (Tello and Forneck 2018). This developmental phase encompasses a period of differential growth rate of rachis structures, which is accelerated during the development of loose clusters (Richter et al. 2017) compared to compact bunches. Gene regulation was studied during three seasons in the samples from three different environments. This approach should allow identifying CA-associated genes that work comprehensively, independently from season and vineyard location.

This study revealed 15 genes that were differentially expressed between loosely and compactly clustered ‘Pinot Noir’ clones under all different environmental conditions. The regulation of these genes was primarily related to cluster architecture (Fig. 5). As expected, it was partially affected also by environmental and experimental fluctuations to various extents (Fig. 6).

At the early stage of BBCH57, the expression of *VvGRF4* was already higher in the loosely clustered clones than in compact and mixed berried clones. A subtle modulation was observed in the genes *VIT_04s0008g01100* (*CYPP711A1*-*like*) and *VIT_18s0001g03160* (*WAT1*-*like*) at this early point. These two genes are members of cluster I of the regulatory groups at the later stage BBCH71. They maintained expression changes at fruit set, with an explicit down-regulation in loosely clustered clones. *VIT_18s0001g03160* is annotated as a *WAT1*-like (‘walls are thin’) encoding gene, a vacuolar transporter of auxin characterized in *Arabidopsis* (Ranocha et al. 2013). The gene *VIT_04s0008g01100* encodes a homolog to cytochrome P450 711A1, a monooxygenase involved in the metabolism of strigolactones (conversion of carlactone to carlactonic acid). Its function has been identified in the *MAX1* mutation in Arabidopsis, which shows increased axillary growth. MAX1 suppresses shoot branching in *Arabidopsis* (Abe et al. 2014). The findings here indicate additional or diversified functions of this gene in grapevine. The cluster I genes with down-regulation in loose clusters further encompass *VIT_08s0007g01370* (*DIR1*-*like*) and *VIT_18s0001g04890* (*SULTR2*-*like*), annotated as a putative lipid transfer protein resp. a sulfate transporter. The genes *VIT_18s0001g04890* and *VIT_18s0001g03160* have also been described to be repressed in ‘Garnacha Tinta’ clones with larger berries (Grimplet et al. 2017). Homologs of *DIR1* have been implicated in long-distance signal transduction during systemic acquired resistance in plant–pathogen interactions (Shah and Zeier 2013). Its transcript reduction in the context of emerging loose cluster architecture is a new aspect. Hypothetically, it may have a role in the transmission of growth-related cellular signals.

Besides the gene encoding *VvGRF4* that was definitely higher expressed in the LCC-type PN clones at BBCH71, expression of the transcription factor-like gene encoding *PRE6* (*VIT_01s0026g02030*) was significantly enhanced in LCCs. PRE6 belongs to the atypical bHLH transcription factor class with no direct DNA binding ability that mediates auxin, brassinosteroid and light signaling and affects photomorphogenesis. A homolog from rice called *ILI1* (increased lamina inclination 1) increased cell elongation (Zhang et al. 2009). Cell elongation may well contribute to important cluster features such as rachis length and shoulder length.

Genes with autonomous up-regulation in LCCs included *VIT_17s0000g05000*. This gene encodes a SEPALLATA1-like developmental regulator. It has probable transcription factor function and is part of the network that regulates flower development in *Arabidopsis* where it prevents indeterminate growth of the flower meristem (Pelaz et al. 2000). Recently, Palumbo et al. (2019) reported *VIT_17s0000g05000* as homeotic gene associated with whorl differentiation in grapevine during the period of pre-anthesis on to post-fertilization. A functional role of *SEP1*-*like* is supported by data available in a transcriptomic atlas derived from spatial–temporal gene expression studies on the grapevine cultivar ‘Corvina’ (Fasoli et al. 2012). In this study, growing rachis tissue showed up-regulation of *VIT_17s0000g05000*, whereas its expression was close to the reference tissue (mesocarp at BBCH77) in tendrils, seed, roots and mature rachis tissue.

In addition to auxin transport functions (*VIT_18s0001g03540, LAX3*-*like*) and auxin homeostasis [*VIT_18s0001g11160, MIZU*-*KUSSEL1* (Moriwaki et al. 2011)], further genes with up-regulation, particularly in loosely clustered PN clones, encompass functions involved in cell wall extension (*VIT_17s0053g00990, EXPA1*-*like*), cell size (*VIT_01s0127g00870, PG1*-*like*) and cell division (*VIT_01s0010g02430, MAD2*). The gene *VIT_17s0053g00990* encodes α-expansin that was found up-regulated in rapidly growing grape berries and permits to enlarge cell size by loosening the fibrillar net in plant cell walls (Suzuki et al. 2015).

In a previous genetic study, QTL clusters associated with loose bunch architecture were localized in a CA segregating population from a cross of ‘Calardis Musqué (formerly named GF.GA-47-42) × ‘Villard Blanc’ (Richter et al. 2019). Arrays of overlapping QTL regions were found on seven chromosomes, including chromosome 1 and 17. Interestingly, the three genes *VIT_01s0026g02030 (PRE6)*, *VIT_17s0000g05000* (S*EP1*-like), and *VIT_17s0053g00990* (*EXPA1*-like), associated with cluster architecture characteristics found here for PN clones, are located in QTL areas. Two of them code for transcription factors that may have a comprehensive function, which needs to be further investigated.

Furthermore, 16 selected individuals from this cross population exhibiting extreme phenotypes for pedicel and rachis lengths were included in the gene expression study. The aim was to check the differential gene regulation of the 15 CA-related genes found in PN in this genetically completely different sample set. Indeed, the expression level of the gene encoding transcription factor *VvPRE6* and six more genes (homologs of *CYP711A1*-*like*, *Mizu*-*Kussel1*, *DIR1*, *WAT1*, *EXPA1* and *PG1*-*like*, Fig. 7a) was significantly linked to extreme CA phenotypes in this divergent germplasm. A corresponding result was obtained in the loosely clustered reference varieties ‘Uva Rara’ and ‘Prosecco’ (Fig. 7a, b). Particularly, the three genes encoding transcription factor PRE6 and the cell wall-related functions EXPA1-like and PG1-like exhibit increased expression levels in loosely clustered plants of diverse genetic background, especially in relation to pedicel length (Fig. 7a). Quite in contrast, the role of *VvGRF4* is specific for the ‘Pinot’ clones, as also inferred from sequencing studies that show the absence of the mutated microRNA binding site in the OIV reference varieties (Rossmann et al. 2019).

This study thus revealed a set of genes with wide relevance for loosely clustered grapevines. These genes enclose components of auxin transport and homeostasis (*WAT1, AUX1*, *Mizu*-*Kussel1*), cell wall structure and loosening (*PG1*, *EXPA1*), in addition to strigolactone metabolism (*CY711A1*, *MAX1*) and the regulatory transcription factor PRE6. These genes deserve further investigation. This novel knowledge facilitates development of gene-targeted markers of loose cluster types for grapevine breeding.

## Conclusions

This study revealed 15 genes with differential gene expression between loosely and compactly clustered PN clones, independently from year and location (or any other environmental variation encountered). It confirmed the role of *VvGRF4* in the control of cluster architecture in ‘Pinot Noir.’ It newly identified two more transcription factor genes, encoding a *SEPALLATA1* homolog and a homolog of *PRE6*, that are more active in the loosely clustered than in the compact bunch type clones. Compared to the recent literature, these regulator genes may have new or additional functions in affecting the structure of the ‘Pinot Noir’ grapevine bunch. Furthermore, genes involved in auxin metabolism, cellular growth and transport were found to be regulated. A gene homolog of CYP711A1, encoding an enzyme of strigolactone metabolism, was also involved. Strigolactones function as shoot branching inhibitors (Gomez-Roldan et al. 2008). This gene is repressed in loose clusters, possibly releasing some inhibition, and thus seems to contribute to the loose-clustered phenotype in grapes.

These results were confirmed for seven genes in completely different genetic backgrounds: the transcription factor gene *PRE6* and six genes related to auxin metabolism, cell wall loosening and strigolactones. They improve the basic knowledge on grapevine cluster phenotype.

This study revealed several major regulators of cluster architecture in ‘Pinot Noir’ and other grapevines, which deserve further attention and functional studies. Future investigation will show if they are applicable as molecular tools for breeding of advantageous loosely clustered grapevine cultivars with improved resilience to *Botrytis cinerea*.

## Electronic supplementary material

Below is the link to the electronic supplementary material.

Supplementary material 1 (DOCX 21 kb)

Supplementary material 2 (DOCX 19 kb)

Supplementary material 3 (DOCX 18 kb)

Supplementary material 4 (DOCX 19 kb)

Supplementary material 5 (DOCX 29 kb)

Supplementary material 6 (PDF 627 kb)

Supplementary material 7 (XLSX 135 kb)

Supplementary material 8 (DOCX 22 kb)

Supplementary material 9 (XLSX 18 kb)

Supplementary material 10 (XLSX 12 kb)

Supplementary material 11 (XLSX 128 kb)
