# Supplementary material for: Differential expression of transcription factor- and further growth-related genes correlates with contrasting cluster architecture in Vitis vinifera ‘Pinot Noir’ and Vitis spp. genotypes
Source: Theor Appl Genet. 2020 Aug 18;133(12):3249–72. doi: 10.1007/s00122-020-03667-0 (PMC7567691; doi:10.1007/s00122-020-03667-0)
Supplement: Supplementary file 1 — Supplementary material 1 (DOCX 21 kb) [file 122_2020_3667_MOESM1_ESM.docx]

**Online resource 1** SSR marker analysis for ‘Pinot Noir’clones at three locations

|  | **VMC3a9_1** | **VMC3a9_2** | **VMC5g7_1** | **VMC5g7_2** | **VMC8g6_1** | **VMC8g6_2** | **VrZAG79_1** | **VrZAG79_2** | **VVS2_1** | **VVS2_2** | **VVMD32_1** | **VVMD32_2** |
| --- | --- | --- | --- | --- | --- | --- | --- | --- | --- | --- | --- | --- |
| PN Reference JKI | 72 | 78 | 190 | 200 | 147 | 169 | 237 | 243 | 137 | 152 | 242 | 274 |
| En777_B_1 | 72 | 78 | 190 | **218** | 147 | 169 | 237 | 243 | 137 | 152 | 242 | 274 |
| En777_B_2 | 72 | 78 | 190 | **218** | 147 | 169 | 237 | 243 | 137 | 152 | 242 | 274 |
| En777_H_1 | 72 | 78 | 190 | **218** | 147 | 169 | 237 | 242 | 137 | 152 | 242 | 274 |
| En777_H_2 | 72 | 78 | 190 | **218** | 147 | 169 | 237 | 243 | 137 | 152 | 242 | 274 |
| FkCL_H_1 | 72 | 78 | 190 | 200 | 147 | 169 | 237 | 243 | 137 | 152 | 242 | 274 |
| FkCL_H_2 | 72 | 78 | 190 | 200 | 147 | 169 | 237 | 243 | 137 | 152 | 242 | 274 |
| FkCL_P_1 | 72 | 78 | 190 | 200 | 147 | 169 | 237 | 243 | 137 | 152 | 242 | 274 |
| FkCL_P_2 | 72 | 78 | 190 | 200 | 147 | 169 | 237 | 243 | 137 | 152 | 242 | 274 |
| FkCh_B_1 | 72 | 78 | 190 | 200 | 147 | 169 | 237 | 243 | 137 | 152 | 242 | 274 |
| FkCh_B_2 | 72 | 78 | 190 | 200 | 147 | 169 | 237 | 243 | 137 | 152 | 242 | 274 |
| FkCh_H_1 | 72 | 78 | 190 | 200 | 147 | 169 | 237 | 243 | 137 | 152 | 242 | 274 |
| FkCh_H_2 | 72 | 78 | 190 | 200 | 147 | 169 | 237 | 243 | 137 | 152 | 242 | 274 |
| FkCh_P_1 | 72 | 78 | 190 | 200 | 147 | 169 | 237 | 243 | 137 | 152 | 242 | 274 |
| FkCh_P_2 | 72 | 78 | 190 | 200 | 147 | 169 | 237 | 243 | 137 | 152 | 242 | 274 |
| Fr12L_B_1 | 72 | 78 | 190 | 200 | 147 | 169 | 237 | 243 | 137 | 152 | 242 | 274 |
| Fr12L_B_2 | 72 | 78 | 190 | 200 | 147 | 169 | 237 | 243 | 137 | 152 | 242 | 274 |
| Fr12L_H_1 | 72 | 78 | 190 | 200 | 147 | 169 | 237 | 243 | 137 | 152 | 242 | 274 |
| Fr12L_H_2 | 72 | 78 | 190 | 200 | 147 | 169 | 237 | 243 | 137 | 152 | 242 | 274 |
| Fr13L_B_1 | 72 | 78 | 190 | 200 | 147 | 169 | 237 | 243 | 137 | 152 | 242 | 274 |
| Fr13L_B_2 | 72 | 78 | 190 | 200 | 147 | 169 | 237 | 243 | 137 | 152 | 242 | 274 |
| Fr13L_H_1 | 72 | 78 | 190 | 200 | 147 | 169 | 237 | 243 | 137 | 152 | 242 | 274 |
| Fr13L_H_2 | 72 | 78 | 190 | 200 | 147 | 169 | 237 | 243 | 137 | 152 | 242 | 274 |
| Fr1801_B_1 | 72 | 78 | 190 | 200 | 147 | 169 | 237 | 243 | 137 | 152 | 242 | 274 |
| Fr1801_B_2 | 72 | 78 | 190 | 200 | 147 | 169 | 237 | 243 | 137 | 152 | 242 | 274 |
| Fr1801_H_1 | 72 | 78 | 190 | 200 | 147 | 169 | 237 | 243 | 137 | 152 | 242 | 274 |
| Fr1801_H_2 | 72 | 78 | 190 | 200 | 147 | 169 | 237 | 243 | 137 | 152 | 242 | 274 |
| Gm186_H_1 | 72 | 78 | 190 | **218** | 147 | 169 | 237 | 243 | 137 | 152 | 242 | 274 |
| Gm186_H_2 | 72 | 78 | 190 | **218** | 147 | 169 | 237 | 243 | 137 | 152 | 242 | 274 |
| Gm1-86_P_1 | 72 | 78 | 190 | **218** | 147 | 169 | 237 | 243 | 137 | 152 | 242 | 274 |
| Gm1-86_P_2 | 72 | 78 | 190 | **218** | 147 | 169 | 237 | 243 | 137 | 152 | 242 | 274 |
| Gm18_H_1 | 72 | 78 | 190 | 200 | 147 | 169 | 237 | 243 | 137 | 152 | 242 | 274 |
| Gm18_H_2 | 72 | 78 | 190 | 200 | 147 | 169 | 237 | 243 | 137 | 152 | 242 | 274 |
| Gm20-13_B_1 | 72 | 78 | 190 | 200 | 147 | 169 | 237 | 243 | 137 | 152 | 242 | 274 |
| Gm20-13_B_2 | 72 | 78 | 190 | 200 | 147 | 169 | 237 | 243 | 137 | 152 | 242 | 274 |
| Gm20-13_H_1 | 72 | 78 | 190 | 200 | 147 | 169 | 237 | 243 | 137 | 152 | 242 | 274 |
| Gm20-13_H_2 | 72 | 78 | 190 | 200 | 147 | 169 | 237 | 243 | 137 | 152 | 242 | 274 |
| Gm20-13_P_1 | 72 | 78 | 190 | 200 | 147 | 169 | 237 | 243 | 137 | 152 | 242 | 274 |
| Gm20-13_P_2 | 72 | 78 | 190 | 200 | 147 | 169 | 237 | 243 | 137 | 152 | 242 | 274 |
| WeM171_P_1 | 72 | 78 | 190 | 200 | 147 | 169 | 237 | 243 | 137 | 152 | 242 | 274 |
| WeM171_P_2 | 72 | 78 | 190 | 200 | 147 | 169 | 237 | 243 | 137 | 152 | 242 | 274 |
| WeM1_H_1 | 72 | 78 | 190 | 200 | 147 | 169 | 237 | 243 | 137 | 152 | 242 | 274 |
| WeM1_H_2 | 72 | 78 | 190 | 200 | 147 | 169 | 237 | 243 | 137 | 152 | 242 | 274 |
| WeM242_H_1 | 72 | 78 | 190 | 200 | 147 | 169 | 237 | 243 | 137 | 152 | 242 | 274 |
| WeM242_H_2 | 72 | 78 | 190 | 200 | 147 | 169 | 237 | 243 | 137 | 152 | 242 | 274 |
