# Supplementary material for: Differential expression of transcription factor- and further growth-related genes correlates with contrasting cluster architecture in Vitis vinifera ‘Pinot Noir’ and Vitis spp. genotypes
Source: Theor Appl Genet. 2020 Aug 18;133(12):3249–72. doi: 10.1007/s00122-020-03667-0 (PMC7567691; doi:10.1007/s00122-020-03667-0)
Supplement: Supplementary file 2 — Supplementary material 2 (DOCX 19 kb) [file 122_2020_3667_MOESM2_ESM.docx]

Online resource 2 Weather recording stations, plant protection schedules, average climate conditions and plant vigor at three trial fields

Average air temperature and precipitation were recorded with the nearest weather stations to the trial field region and at comparable latitude during the period of April to September. Vegetative vigor was estimated with the weight of the pruned wood per vine.

| Trial field location  (Trial field management) | Weather station (Identifier) | latitude over zero: | Trial field established / vine spacing | Plant protection schedule | Season | Average Air temp.  [°C] | Average precipitation  Apr.-Sept. [mm/m^2^]¶ | Pruning wood weight  [kg] |
| --- | --- | --- | --- | --- | --- | --- | --- | --- |
| Baden  48°07'15.9"N 7°37'06.0“E  (integrated) | König-schaffhausen (84) | 185 m | 1997  2.0m*1.1m | BBCH 17-65 sulfur, synthetic fungicides  BBCH 65-81 synthetic fungicides  every 10-12 days | 2015  2016  2017 | 12.0  11.2  11.7 | 47.1  67.9  53.5 | 1.176^a^  1.096^a^  - |
| Hesse  49°37'28.7"N 8°38'54.0“E  (integrated) | Hirschberg (135) | 100 m | 1995  1.8m*1.0m | BBCH 17-65 sulfur, synthetic fungicides  BBCH 65-81 synthetic fungicides  every 10-12 days | 2015  2016  2017 | 12.0  11.4  11.0 | 50.5  64.9  73.1 | 0.720^b^  0.795^b^  - |
| Palatinate  49°13'07.8"N 8°02'40.5“E  (organic) | Siebeldingen (88) | 192 m | 2003  2.0m*1.0m | BBCH 17-81 copper, sulfur,  BBCH 79-85 copper, carbonates  every 7 days | 2015  2016  2017 | 11.7  10.8  11.0 | 36.0  48.5  53.3 | 0.408^c^  0.504^c^  - |
| Letters given in superscript form indicate significant differences between measurement records according to ANOVA α=0.05 | | | | | | | | |
