# Supplementary material for: Differential expression of transcription factor- and further growth-related genes correlates with contrasting cluster architecture in Vitis vinifera ‘Pinot Noir’ and Vitis spp. genotypes
Source: Theor Appl Genet. 2020 Aug 18;133(12):3249–72. doi: 10.1007/s00122-020-03667-0 (PMC7567691; doi:10.1007/s00122-020-03667-0)
Supplement: Supplementary file 3 — Supplementary material 3 (DOCX 18 kb) [file 122_2020_3667_MOESM3_ESM.docx]

Online resource 3 Sampling schedule for gene expression experiments.

| Sampling schedule | | | | | |
| --- | --- | --- | --- | --- | --- |
| Location | BBCH | 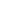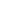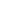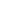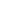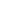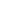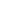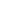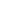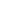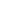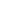  Season | Day of year | Sampling | °CDD start at BBCH09 |
| P | 57 | 2015 | 152 | 01.06.2015 | 421.2 |
| P | 57 | 2016 | 161 | 09.06.2016 | 415.69 |
| P | 57 | 2017 | 154 | 03.06.2017 | 416.65 |
| H | 57 | 2015 | 149 | 29.05.2015 | 398.27 |
| H | 57 | 2016 | 157 | 05.06.2016 | 447.21 |
| H | 57 | 2017 | 152 | 01.06.2017 | 401.87 |
| B | 57 | 2015 | 146 | 31.05.2015 | 390.33 |
| B | 57 | 2016 | 159 | 07.06.2016 | 422.07 |
| B | 57 | 2017 | 150 | 30.05.2017 | 420.15 |
| P | 71 | 2015 | 177 | 26.06.2015 | 714.83 |
| P | 71 | 2016 | 186 | 04.07.2016 | 708.59 |
| P | 71 | 2017 | 177 | 26.06.2017 | 712.11 |
| H | 71 | 2015 | 176 | 25.06.2015 | 725.96 |
| H | 71 | 2016 | 178 | 26.06.2016 | 703.37 |
| H | 71 | 2017 | 171 | 22.06.2017 | 677.38 |
| B | 71 | 2015 | 170 | 19.06.2015 | 698.01 |
| B | 71 | 2016 | 182 | 30.06.2016 | 712.93 |
| B | 71 | 2017 | 171 | 20.06.2017 | 711.26 |

Rachis samples (three unrelated biological repeats) were taken twice, at pre bloom (phenological stage BBCH57) and at past bloom (BBCH71; see Figure 3) at the three locations Palatinate (P), Hesse (H) and Baden (B) during the seasons 2015-2017. The sampling dates ranged over up to 16 days due to the targets for cumulated degree day (CDD) sum (400° BBCH57 and 700° BBCH71) for the phenological stages according to Molitor et al. (2014)
