# Supplementary material for: Differential expression of transcription factor- and further growth-related genes correlates with contrasting cluster architecture in Vitis vinifera ‘Pinot Noir’ and Vitis spp. genotypes
Source: Theor Appl Genet. 2020 Aug 18;133(12):3249–72. doi: 10.1007/s00122-020-03667-0 (PMC7567691; doi:10.1007/s00122-020-03667-0)
Supplement: Supplementary file 4 — Supplementary material 4 (DOCX 19 kb) [file 122_2020_3667_MOESM4_ESM.docx]

Online resource 4 Phenotypic measurements recorded during four seasons on selected F1 individuals of the cross population (‘Calardis Musqué’ × ‘Villard Blanc’).

Mean of pedicel lengths and rachis lengths measured at selected F1 individuals recorded over four seasons. The selected genotypes showed distinct short resp. long pedicel- and rachis lengths.

n= number of independently sampled clusters per genotype, for each cluster ten pedicels were measured

| **Pheno-type** | **Genotype** | **Pedicel length**  **[cm]** | | | | **Pheno- type** | **Genotype** | **Rachis length**  **[cm]** | | | |
| --- | --- | --- | --- | --- | --- | --- | --- | --- | --- | --- | --- |
|  |  | **2013**  (n=12) | **2014**  (n=3) | **2015**  (n=6) | **2016**  (n=6) |  |  | **2013**  (n=12) | **2014**  (n=3) | **2015**  (n=6) | **2016**  (n=6) |
| PED max | 89-30-212 | 0.63 | 0.58 | 0.73 | 0.71 | RL max | 89-30-405 | 16.6 | 20.27 | 22.79 | 25.11 |
| PED max | 89-30-294 | 0.63 | 0.53 | 0.7 | 0.64 | RL max | 89-30-484 | 13.6 | 18.71 | 22.13 | 23.85 |
| PED max | 89-30-354 | 0.67 | 0.53 | 0.67 | 0.74 | RL max | 89-30-503 | * | 22.65 | 22.47 | 29.25 |
| PED max | 89-30-380 | 0.66 | 0.64 | 0.65 | 0.67 | RL max | 89-30-059 | 16.23 | 23.61 | 22.45 | 25.21 |
| PED min | 89-30-194 | 0.49 | 0.27 | 0.39 | 0.49 | RL min | 89-30-241 | * | * | 9.63 | 7.74 |
| PED min | 89-30-558 | 0.48 | 0.25 | 0.39 | 0.4 | RL min | 89-30-647 | 9.34 | 12.21 | 10.93 | 9.18 |
| PED min | 89-30-594 | 0.43 | 0.35 | 0.4 | 0.47 | RL min | 89-30-680 | 11.24 | 7.29 | 12.01 | 15.06 |
| PED min | 89-30-598 | 0.34 | 0.29 | 0.39 | 0.45 | RL min | 89-30-052 | 9.93 | 14.26 | 14.15 | 14.9 |

T-test results and descriptive values for the measurements of pedicel lengths and rachis lengths of the selected F1 hybrids

PED max = four individuals with extreme long pedicel length,

PED min = four individuals with extreme short pedicel length

RL max = four individuals with extreme long rachis length

RL min = four individuals with extreme short rachis length)

SEM = Standard error of the mean

| T-test (*p*-value) | PED max | PED min | RL max | RL min |
| --- | --- | --- | --- | --- |
|  | *p*-value = (5.45E-11) *df*(27) | | *p*-value = (1.24E-08) *df*(23) | |
| Mean | 0.64 | 0.39 | 21.66 | 11.27 |
| SEM | 0.015 | 0.019 | 1.035 | 0.692 |
| Minimum | 0.53 | 0.25 | 13.6 | 7.29 |
| Maximum | 0.74 | 0.49 | 29.25 | 15.06 |
