# Supplementary material for: Differential expression of transcription factor- and further growth-related genes correlates with contrasting cluster architecture in Vitis vinifera ‘Pinot Noir’ and Vitis spp. genotypes
Source: Theor Appl Genet. 2020 Aug 18;133(12):3249–72. doi: 10.1007/s00122-020-03667-0 (PMC7567691; doi:10.1007/s00122-020-03667-0)
Supplement: Supplementary file 5 — Supplementary material 5 (DOCX 29 kb) [file 122_2020_3667_MOESM5_ESM.docx]

**Online resource 5** Primers for the quantitative Real Time amplification of candidate genes and reference genes. Primers used for the reference gene amplification are highlighted in grey.

^1^Annotation based on RNA-Seq results reported in (Rossmann et al. 2020). The *Vitis* gene annotations for candidate genes based on literature reporting data for rice and tomato were retrieved with their protein sequence from the NCBI Gene Bank (https://www.ncbi.nlm.nih.gov/nuccore). This sequence was then used for an orthologue search restricted to orthologs in *Vitis vinifera* with the ‘hierarchical catalogue of orthologs’ (https://www.orthodb.org) (Kriventseva et al. 2019).

^2^Amplicon length of the product based on the reference genome PN40024 assembly version 12x v2 (Canaguier et al. 2017)

| ^1^Gene ID V1 | Gene ID costV3 | Forward sequence 5' -->3' | Reverse sequence 5' -->3' | ^2^Amp [bp] | Bibliography |
| --- | --- | --- | --- | --- | --- |
| *VIT_00s0313g00070* | *Vitvi07g01441* | AGGTTGAGCAAGGAAGTTGCA | CTCGGCTCAATCCAGCTTCA | 127 | Jiang et al. 2012 |
| *VIT_01s0010g01810* | *Vitvi01g01457* | TCGCCGTTGTCCGAGTTT | ACTTCCACTCCACCACCT | 151 | Rossmann et al. 2020 |
| *VIT_01s0010g02430* | *Vitvi01g01534* | CAAGATGAGGGTGTTAAATCGT | ACCTCATTTGTTGCCTTGCT | 119 | Rossmann et al. 2020 |
| *VIT_01s0011g06410* | *Vitvi01g00553* | CTCCATGCGGGTCCTTGT | GTGCGTTGGTTTCTGGGATT | 108 | Jiang et al. 2012 |
| *VIT_01s0026g02030* | *Vitvi01g00964* | AAGCCAAAAGCGCAGACA | GCAATAGGCGCTCCGACAA | 118 | Zhang et al. 2009 |
| *VIT_01s0127g00260* | *Vitvi01g00698* | GGCGCGCAAGAAGATCAGAGA | CCCACGCTTGCCAAATAACAT | 199 | Rossmann et al. 2020 |
| *VIT_01s0127g00710* | *Vitvi01g00733* | CCCACCTCCTTTATGACCGCTA | CAAGAAAATCCTCCATCAACCGT | 232 | Rossmann et al. 2020 |
| *VIT_01s0127g00870* | *Vitvi01g00747* | AGGCGTCTTTGCTTCGGTATT | CGCATTTTGAGCGGCAAGT | 134 | Rossmann et al. 2020 |
| *VIT_01s0146g00400* | *Vitvi01g01733* | TCCCACTCCGACACCACCTT | TCTTCCTTGGCTTTCTTGCCGTTT | 131 | Rossmann et al. 2020 |
| *VIT_01s0146g00480* | *Vitvi01g02293* | CCGCCATGGAACTTGATTTCT | GCGAACGGCGGATTATTCT | 196 | Rossmann et al. 2020 |
| *VIT_02s0012g00990* | *Vitvi02g00666* | GCTGCCACACCTTACTCAT | ATGTACTTACCCCAACAGATGTC | 204 | Rossmann et al. 2020 |
| *VIT_02s0012g01380* | *Vitvi02g00702* | GAGACTCCGGCCACCAACAA | GCCCAGCCTTCACCACATTT | 128 | Rossmann et al. 2020 |
| *VIT_02s0012g01400* | *Vitvi02g00704* | CCTCGATTCATTCCGCTTCT | CGGCTGCTGATGCTTCTT | 85 | Rossmann et al. 2020 |
| *VIT_02s0025g03010* | *Vitvi02g00276* | GGCTGCGAGAGAGTCGTTAAA | ACCTTTTCCATCCCCAGATCCA | 83 | Rossmann et al. 2020 |
| *VIT_02s0025g03140* | *Vitvi02g01375* | CCCGGTTTGACATTTCTCAT | CCTCTTGCACTTCGAATCCT | 68 | Rossmann et al. 2020 |
| *VIT_02s0025g03180* | *Vitvi02g00287* | CAACATGGTCCCTGCAATC | GGTTGGAGATGGAGCTTCTG | 190 | Rossmann et al. 2020 |
| *VIT_02s0025g04340* | *Vitvi02g01409* | CCGAGTGAAATAAGGCATGT | ATAATTGAGGAGGGCTCACA | 41 | Rossmann et al. 2020 |
| *VIT_02s0025g04660* | *Vitvi02g00429* | TTGACTGCTGCTCTTGTGCTT | CCACTCCCAAAAACAGAACCTT | 133 | Rossmann et al. 2020 |
| *VIT_02s0025g04720* | *Vitvi02g00435* | CCCTGAAGACAAGCGCGATA | GGGTACCATGTTGTGGAGGATGAAG | 298 | Rossmann et al. 2020 |
| *VIT_02s0154g00320* | *Vitvi02g00532* | CCTTCTCCTTGCCCTAAACCT | GGTGGCTTTTTGTGGTGGTTTTT | 102 | Rossmann et al. 2020 |
| *VIT_02s0154g00380* | *Vitvi02g01443* | CAGCCTCCTCTACAACCT | CTGCTGCTGCTTCTTCTT | 130 | Rossmann et al. 2020 |
| *VIT_02s0241g00030* | *Vitvi02g01424* | CTTCAGTCTTCACCTACTGTGA | AGAAGCTTCTTTTGATACCGATAG | 70 | Rossmann et al. 2020 |
| *VIT_03s0097g00700* | *Vitvi03g00860* | GGCCTTATGGGGAGAACCTT | TGCCGCAGTGCCTGTAAA | 56 | Rossmann et al. 2020 |
| *VIT_04s0008g00180* | *Vitvi04g00009* | CCCTGGACTGTTTCTGTTGCT | AGGACTGCTGGGGGCAAAA | 128 | Rossmann et al. 2020 |
| *VIT_04s0008g00370* | *Vitvi04g00029* | CAAGCAAGGAGAGCCAGACA | CCCGTCACAAGCTCAAGCAA | 133 | Rossmann et al. 2020 |
| *VIT_04s0008g01100* | *Vitvi04g00091* | CCCCTTGATGGCCAAGTAT | GGAGAGGGGATGCTGAGAT | 197 | Rossmann et al. 2020 |
| *VIT_04s0008g01810* | *Vitvi04g00155* | GCTGCAGATTGAGGTGGTT | GTCTGTTCGCCCTGGAAT | 149 | Rossmann et al. 2020 |
| *VIT_04s0008g01910* | *Vitvi04g00164* | TCTCCCTCTCCCTCGTCTTC | CATCCTCACCCCCCACTTCA | 210 | Rossmann et al. 2020 |
| *VIT_04s0008g02900* | *Vitvi04g00256* | GGGAGGAATTGAAGGCTATGG | GCACCAATGCGCAGCAAA | 162 | Rossmann et al. 2020 |
| *VIT_04s0008g02920* | *Vitvi04g00259* | GTGGCTCCCCAGTTAGTGAT | ACCCACCGACAGTTCTTTTG | 145 | Rossmann et al. 2020 |
| *VIT_04s0008g04050* | *Vitvi04g00350* | CCTCACACTCCCATGCCCAAA | CCCAAACAAAAAGCAGCAGCAGAA | 89 | Rossmann et al. 2020 |
| *VIT_04s0008g04200* | *Vitvi04g00361* | CGAGAGTGCCCAAGAGGTT | CGCATGACCCTGGCAGAA | 108 | Rossmann et al. 2020 |
| *VIT_04s0008g05150* | *Vitvi04g01894* | TCTCGCCCAAGGGGTTTT | CTGAAACACTCCATCCTGCTT | 145 | Rossmann et al. 2020 |
| *VIT_04s0008g05770* | *Vitvi04g00512* | GGCCGGAAAGGGAGGTTAT | CGCCAGCCGACTTCAAGA | 88 | Rossmann et al. 2020 |
| *VIT_04s0008g05830* | *Vitvi04g00517* | GTCTCAGATCGCGTCATTGT | TTGTGGACAGCTCCTGCTT | 93 | Rossmann et al. 2020 |
| *VIT_04s0008g06670* | *Vitvi04g00602* | CCCAATCCGATTCTCTCAACAA | CCCTCCTCACCTTCAACAC | 123 | Rossmann et al. 2020 |
| *VIT_04s0023g03070* | *Vitvi04g01426* | GGACCTAAGCTGGAACAAG | CACCGTTGCAGGAATCTT | 118 | Jiang et al. 2012 |
| *VIT_04s0069g00790* | *Vitvi04g00736* | ATCCCAGCAAAGACATCAGT | AAACAGAACCAGGCCCAAGA | 212 | Rossmann et al. 2020 |
| *VIT_04s0079g00260* | *Vitvi04g00836* | ACCACAAGCCTGCAATTTT | GGCTCTGACCTCAAGGTT | 112 | Rossmann et al. 2020 |
| *VIT_07s0031g01850* | *Vitvi07g01861* | TGGGCAGCTAGGAGGAAGATT | GTGGGGGATGCAGTTATGGT | 75 | Jiang et al. 2012 |
| *VIT_08s0007g01310* | *Vitvi08g01277* | ATGGGAAGAGCTGGTTTGG | AGCGGCTAGTGTTCAAATCC | 42 | Rossmann et al. 2020 |
| *VIT_08s0007g01320* | *N.A.* | GGGGCCGATTCTCAACAGT | ACCACCTCATGGACCTTCCT | 142 | Rossmann et al. 2020 |
| *VIT_08s0007g01350* | *Vitvi08g02224* | CTCCTCCTCCTCACGACAGA | CACGCCATCACAGCACTT | 76 | Rossmann et al. 2020 |
| *VIT_08s0007g01360* | *Vitvi08g02225* | AACGCCAAACCAGGGACTACA | ACTCTGACTCTCGCCTTCACT | 60 | Rossmann et al. 2020 |
| *VIT_08s0007g01370* | *Vitvi08g01281* | GGCGGCCAGCGACAAGA | GGCAGCTTGGGTTCTGGAT | 80 | Rossmann et al. 2020 |
| *VIT_08s0040g00040* | *Vitvi08g00880* | GAGGGTCGTCAGGATTTGGA | GCCCTGCACTTACCATCTCTA | 71 | Selim et al. 2012 |
| *VIT_08s0040g01710* | *Vitvi08g01022* | ACTGGATTTGGTGCGACTT | CGTGTGGCATGAGTCTGTT | 117 | Rossmann et al. 2020 |
| *VIT_08s0058g00930* | *Vitvi08g00816* | TCGGACGGGGAAAAGTATGCAA | CCTGGGGCCAACTCTACAAT | 125 | Rossmann et al. 2020 |
| *VIT_08s0058g00990* | *Vitvi08g00823* | TGGGTGCTTCTTTGCTTCGT | CGCCCGCATTCTTTTCACT | 111 | Rossmann et al. 2020 |
| *VIT_09s0070g00470* |  | TGCCAAAAGGGACCTCTGAT | TCGGGAGGAGGAAGAGGAGCTA | 113 | Jiang et al. 2012 |
| *VIT_11s0016g03710* | *Vitvi11g00317* | GTCCGAATCGGCTGCTTGAA | TCGGGTTCCATCGCACTT | 88 | Rossmann et al. 2020 |
| *VIT_12s0059g00190* | *Vitvi12g00342* | CTCCGGCCAGCTCCAACA | GCCCCTACTCTTGCCCTAAAC | 153 | Dal Santo et al. 2013 |
| *VIT_14s0066g01060* | *Vitvi14g01745* | CCACCTACAGAACTCCCAAAA | TATCCCTCCCTAGACCTCCAAT | 158 | Rossmann et al. 2020 |
| *VIT_14s0066g01390* | *Vitvi14g01780* | ATTTGACTCGGGGAAAGCA | TGGCAGCAAGTGACTGATG | 110 | Rossmann et al. 2020 |
| *VIT_14s0083g00410* | *Vitvi14g01273* | CCTTCCCAACCTCCCTTTC | CCTCTCCAACCCCATCATCAC | 173 | Correa et al. 2014 |
| *VIT_14s0108g00700* | *Vitvi14g01952* | AGTGCGAGTGATGAACAGAGA | GGGCTGCTGCGTATAGTG | 184 | Rossmann et al. 2020 |
| *VIT_14s0108g00740* | *Vitvi14g03084* | CGCCATTTCCATGCTTCAC | CAAAACAACACTCGCACACAATC | 139 | Rossmann et al. 2020 |
| *VIT_14s0219g00230* | *Vitvi14g01635* | CCGGCTGGTGGACAGTAT | AGAGATGGTTATGGCGGTGGAT | 140 | Vargas et al. 2013 |
| *VIT_15s0048g01750* | *Vitvi15g00816* | CCACCACTCTCTACCAAACC | CCGACCTTGCCACCTTTCA | 85 | Rossmann et al. 2020 |
| *VvGRF4* | *Vitvi16g00073* | ACCAACCAATCCCAATTCCA | TTCGCCTACCTCGGGTTT | 102 | Rossmann et al. 2020 |
| *VIT_17s0000g02470* | *Vitvi17g00217* | GGTCCCTGCTTCTCAGTCT | TTGCCTGCGCCTGGTTGTA | 121 | Rossmann et al. 2020 |
| *VIT_17s0000g03550* | *Vitvi17g00307* | GGAGAGAGAAAGGCTCGAGTT | AGCATGGAAAGGCGATCAT | 104 | Rossmann et al. 2020 |
| *VIT_17s0000g03750* | *Vitvi17g01407* | ACAGAGAGGGGAGAGCTT | TTGTACCACCTGAGATTTGCT | 159 | Rossmann et al. 2020 |
| *VIT_17s0000g04470* | *Vitvi17g01426* | TCCGCCCTGTGTTCTTCT | AACAACTTTCCGATTCCAGATAC | 60 | Rossmann et al. 2020 |
| *VIT_17s0000g05000* | *Vitvi17g00471* | GTCGCCTTCCTGCTCAATC | CGGGGCCAAATCCATTGT | 151 | Rossmann et al. 2020 |
| *VIT_17s0000g05070* | *Vitvi17g00480* | TCTCTCTCCATAACCTCCCTCAAAC | CCATTAGCGGTGGCAGAAC | 159 | Rossmann et al. 2020 |
| *VIT_17s0000g05570* | *Vitvi17g00533* | GCAGGCTTCCCACTTCAAA | CGCTCATCTTGTCCACCAT | 94 | Rossmann et al. 2020 |
| *VIT_17s0000g07350* | *Vitvi17g00713* | GAGGATGTGCTGAGGATGGA | TGTGGTCGCATAGCCGTTT | 118 | Rossmann et al. 2020 |
| *VIT_17s0000g09190* | *Vitvi17g00906* | AGGGTTCTTGCTGTGGAT | ACACAACTCCCCTAACTTCAC | 66 | Rossmann et al. 2020 |
| *VIT_17s0000g09310* | *Vitvi17g00919* | ACCCCCGATGACTACCTTT | CCCTGTGCTTTTGCTGGAT | 169 | Rossmann et al. 2020 |
| *VIT_17s0000g09470* | *Vitvi17g00936* | AATTGTCACAGCTTCACCCAAAG | CGCGGTCCACTTGGCTTATC | 164 | Rossmann et al. 2020 |
| *VIT_17s0000g09790* | *Vitvi17g00975* | GGGTTGGATGTTTTTGCAAGAT | CCGCCTACTTCGCTTCTTC | 97 | Rossmann et al. 2020 |
| *VIT_17s0000g10430* | *Vitvi17g01598* | TTCTCGTTGAGGGCTATT | CCACAGACTTCATCGGTGACA | 70 | Selim et al. 2012 |
| *VIT_17s0053g00990* | *Vitvi17g01251* | CTTCTATGGCGGGGGTGAT | GCCACAGCTCAACCCATT | 133 | Hoffmann 2015 |
| *VIT_18s0001g03160* | *Vitvi18g00289* | CGCCTTTCGCACTTGTTC | GGAAGCCAAGCACCATTATTTT | 84 | Rossmann et al. 2020 |
| *VIT_18s0001g03540* | *Vitvi18g00310* | GGGCTACCAACATTCTCTACAC | TCCCCAAAAGCCCAATAAACAG | 167 | Rossmann et al. 2020 |
| *VIT_18s0001g04890* | *Vitvi18g00363* | TGTGCCGGTGCCTTCTTT | CCTTCTACGCTGGGCCTAA | 118 | Rossmann et al. 2020 |
| *VIT_18s0001g04910* | *Vitvi18g00365* | ATCTGCGGCTTGCATTCAC | AGCTCCACCCATAAAACCAACA | 128 | Rossmann et al. 2020 |
| *VIT_18s0001g05060* | *Vitvi18g02571* | CAAGCCTCAACTGCTCATAC | CACATCAACACAACCAGTGAAC | 165 | Rossmann et al. 2020 |
| *VIT_18s0001g05800* | *Vitvi18g00414* | GGCATTGACTGGGACCAAAA | CCACCTCTTCTGCATCTCT | 140 | Rossmann et al. 2020 |
| *VIT_18s0001g07340* | *Vitvi18g00510* | CCCGGTCAGCTTATGTTCAT | AGTGTTGGGGGAGAAGGT | 104 | Rossmann et al. 2020 |
| *VIT_18s0001g07460* | *Vitvi18g00517* | GCAGATGAGGGGAGAGGATA | GTGGCGATCTCGGTCATT | 129 | Jiang et al. 2012 |
| *VIT_18s0001g09230* | *Vitvi18g00675* | ACAAGCGATGCCACTACGAA | GGCAGGTTGAGGTCGAAGT | 106 | Rossmann et al. 2020 |
| *VIT_18s0001g09400* | *Vitvi18g00687* | CGGATTGCTGGTTCGTCAT | GTCCTTCGTTGCGTCCTT | 116 | Rossmann et al. 2020 |
| *VIT_18s0001g09510* | *Vitvi18g02657* | AGGGAGGCAGAAGACGATGA | GTCCCAGCCGAGGTATCTGT | 132 | Rossmann et al. 2020 |
| *VIT_18s0001g09910* | *Vitvi18g00730* | CGAAAGAAGCCAACAGCAT | CACCGTTTCTGGCGCATA | 140 | Rossmann et al. 2020 |
| *VIT_18s0001g10130* | *Vitvi18g00755* | CACCCGTGAAGGCAAGTC | CGCCGTCTTTGTCATGTT | 83 | Rossmann et al. 2020 |
| *VIT_18s0001g10610* | *Vitvi18g02683* | AAACATGCCTCGTCATTGGAA | CGCCGTTTTTGTCATGGT | 119 | Rossmann et al. 2020 |
| *VIT_18s0001g10640* | *Vitvi18g02686* | CCGTACGTGCCTAGATTAAAGAA | CCAAGCATCCCCAAATGGAA | 44 | Rossmann et al. 2020 |
| *VIT_18s0001g11160* | *Vitvi18g00842* | GTTCGTTTGGGCTGTGTACT | CTCCTCGTCTGACATTTGCTT | 76 | Rossmann et al. 2020 |
| *VIT_19s0015g00270* | *Vitvi19g02058* | CGGAGAGTGCTGCTGATGAT | GCTTGACTTTTTCGGGTTTTCGT | 149 | Rossmann et al. 2020 |
| *VIT_19s0015g00490* | *Vitvi19g02064* | ACGGAACCGGAGAAGACACT | CCCCATCAGAATCGCCATCT | 108 | Rossmann et al. 2020 |
| *VIT_19s0015g01230* | *Vitvi19g00750* | CGTTGTGGAAATAGCTGTGGAT | AATGGGTGGTGGTGGATTG | 87 | Rossmann et al. 2020 |
| *VIT_19s0015g01890* | *Vitvi19g00928* | CATTCATCACCCCCGTCTCT | ATTCCCACATTCCCCAAACTCA | 93 | Jiang et al. 2012 |
