# Supplementary material for: Differential expression of transcription factor- and further growth-related genes correlates with contrasting cluster architecture in Vitis vinifera ‘Pinot Noir’ and Vitis spp. genotypes
Source: Theor Appl Genet. 2020 Aug 18;133(12):3249–72. doi: 10.1007/s00122-020-03667-0 (PMC7567691; doi:10.1007/s00122-020-03667-0)
Supplement: Supplementary file 6 — Supplementary material 6 (PDF 627 kb) [file 122_2020_3667_MOESM6_ESM.pdf]

Richter et al. Differential expression of three transcription factor- and further growth related genes correlates with contrarious cluster architecture in *Vitis vinifera* 'Pinot Noir' and *Vitis spp.* genotypes

Online resource 6a Effects of trial location and growing season on important cluster architecture sub traits and compactness indices for the 'Pinot Noir' clones Gm20-13 and FkCH, which were the two reference clones that were sampled across all seasons and locations. Means and 95% confidence intervals were estimated with generalized linear models (n = 120).

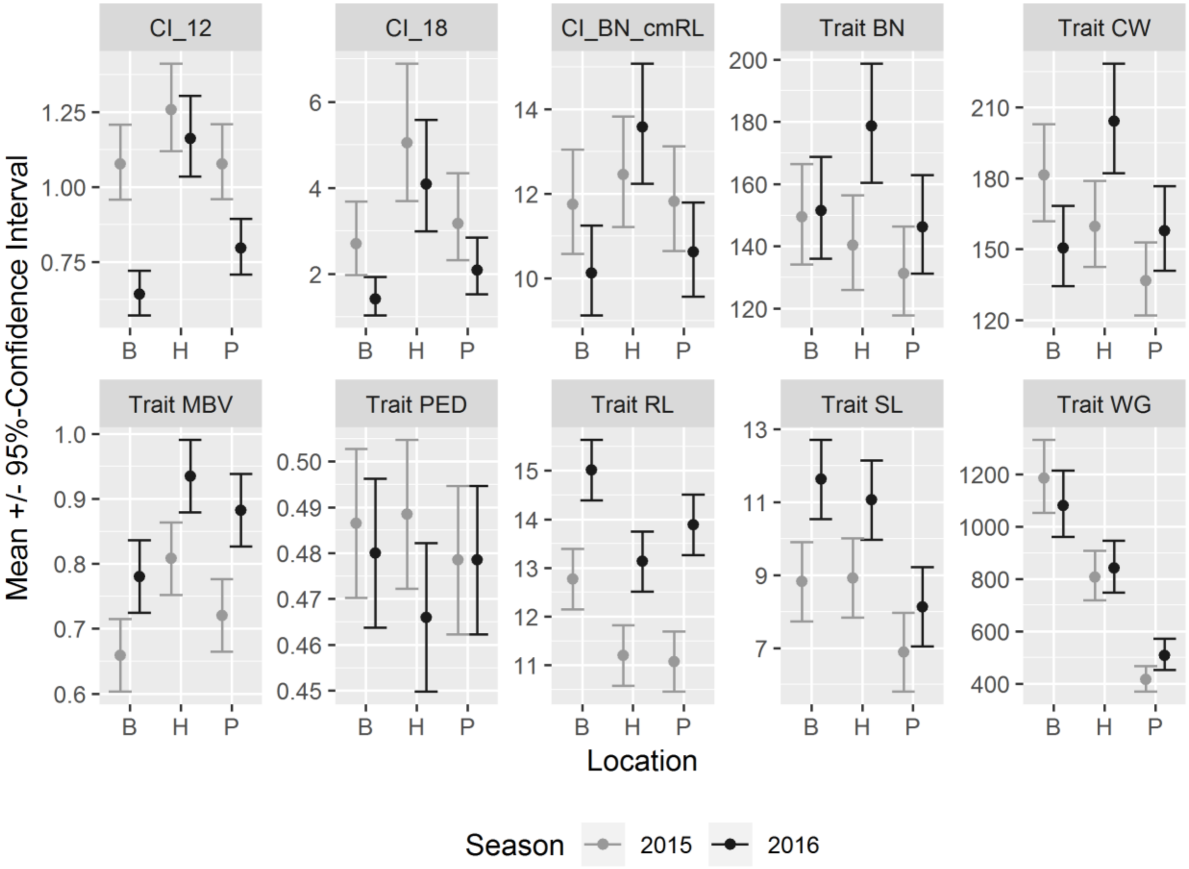

Estimated marginal means for measurements from 2015 and 2016 at three trial fields located in German wine growing regions. Hesse (H) and Palatinate (P) belong to viticulture area A (cool climate). Baden (B) belongs to viticulture area B (moderate climate). For trait abbreviations see table 3.

Online resource 6b ANOVA results for important cluster architecture sub traits and compactness indices for two 'Pinot Noir' clones that were sampled across all seasons and locations. Means and 95% confidence intervals were estimated with generalized linear models.

ANOVA results of a reciprocal design (n = 120) for the measurements of the 'Pinot Noir' clones Gm20-13 and FkCH at all locations and seasons.

P-values for the effects of field location and growing season on cluster architecture sub-traits and compactness indices, obtained from generalized linear models (GLM) with negative binomial (NB) or gamma distribution or ordinary least squares models (OLS) and ANOVA sums of squares type 3 test. For trait abbreviations see table 3.

Trait / Index	Model	Clone	Location	Season	Location: Season
BN	NB GLM	0.000	0.059	0.008	0.130
CW	Gamma GLM	0.000	0.004	0.178	0.002
MBV	OLS	0.000	0.002	0.000	0.868
TBV	NB GLM	0.000	0.000	0.000	0.195
RD	OLS	0.004	0.000	0.133	0.151
RL	OLS	0.019	0.000	0.000	0.436
RW	OLS	0.000	0.006	0.000	0.367
SL	OLS	0.722	0.000	0.000	0.362
PL	OLS	0.125	0.208	0.081	0.101
PED	OLS	0.882	0.746	0.155	0.378
L1I	OLS	0.694	0.168	0.199	0.568
L2I	OLS	0.179	0.274	0.089	0.337
BN_cmRL	Gamma GLM	0.000	0.000	0.188	0.052
CI_12	Gamma GLM	0.000	0.000	0.000	0.000
CI_18	Gamma GLM	0.081	0.000	0.004	0.606
WG	Gamma GLM	0.024	0.000	0.293	0.043

Richter et al. Differential expression of three transcription factor- and further growth related genes correlates with contrarious cluster architecture in *Vitis vinifera* 'Pinot Noir' and *Vitis. spp* genotypes

Online resource 6c ANOVA results for important cluster architecture sub traits and compactness indices for 12 'Pinot Noir' clones that were sampled across all seasons and locations. Means and 95% confidence intervals were estimated with generalized linear models.

ANOVA results for measurements of twelve 'Pinot Noir' clones (n = 400).

P-values for the effects of field location and growing season on cluster architecture sub-traits and compactness indices, obtained from generalized linear models (GLM) with negative binomial (NB) or gamma distribution or ordinary least squares models (OLS) and ANOVA sums of squares type 3 test. For trait abbreviations see table 3.

Trait / Index	Model	Clone	Location	Season	Location: Season
BN	NB GLM	0.056	0.000	0.004	0.001
CW	Gamma GLM	0.000	0.000	0.000	0.000
MBV	OLS	0.000	0.009	0.000	0.000
TBV	NB GLM	0.000	0.000	0.009	0.327
RD	OLS	0.000	0.000	0.009	0.327
RL	OLS	0.000	0.000	0.000	0.244
RW	OLS	0.000	0.000	0.000	0.007
SL	OLS	0.000	0.004	0.000	0.527
PL	OLS	0.000	0.008	0.062	0.107
PED	OLS	0.000	0.662	0.000	0.368
L1I	OLS	0.053	0.864	0.235	0.216
L2I	OLS	0.083	0.159	0.225	0.983
BN_cmRL	Gamma GLM	0.000	0.000	0.043	0.041
CI_12	Gamma GLM	0.000	0.000	0.001	0.000
CI_18	Gamma GLM	0.000	0.000	0.021	0.058
WG	Gamma GLM	0.000	0.000	0.007	0.042
